# Comparative Phytochemical and Biological Evaluation of Walnut Septum and Pellicle Extracts in Chemical, Computational, and Cell-Based Models

**DOI:** 10.3390/ph19101657

**Published:** 2026-10-20

**Authors:** Letiția Mateș, Denisia Pașca, Ionel Fizeșan, Ana-Maria Vlase, Gabriel Marc, Ionuț-Valentin Creștin, Oana Andreea Alexa, Ioan Tomuță, Lorena Filip, Laurian Vlase, Daniela-Saveta Popa

**Affiliations:** 1 Department of Toxicology, Faculty of Pharmacy, Iuliu Hatieganu University of Medicine and Pharmacy, 8 Victor Babeș, 400012 Cluj-Napoca, Romania; micu.letitia@umfcluj.ro (L.M.); ionel.fizesan@umfcluj.ro (I.F.); ionut.vale.crestin@elearn.umfcluj.ro (I.-V.C.); alexaoana9@gmail.com (O.A.A.); dpopa@umfcluj.ro (D.-S.P.); 2 Department of Nutrition and Medical Cosmetics, Faculty of Nursing and Health Sciences, Iuliu Hatieganu University of Medicine and Pharmacy, 23 Gheorghe Marinescu, 400337 Cluj-Napoca, Romania; denisia.pasca@umfcluj.ro; 3 Department of Pharmaceutical Botany, Faculty of Pharmacy, Iuliu Hatieganu University of Medicine and Pharmacy, 8 Victor Babeș, 400012 Cluj-Napoca, Romania; 4 Department of Organic Chemistry, Faculty of Pharmacy, Iuliu Hatieganu University of Medicine and Pharmacy, 8 Victor Babeș, 400012 Cluj-Napoca, Romania; marc.gabriel@umfcluj.ro; 5 Department of Pharmaceutical Technology and Biopharmacy, Faculty of Pharmacy, Iuliu Hatieganu University of Medicine and Pharmacy, 8 Victor Babes, 400012 Cluj-Napoca, Romania; tomutaioan@umfcluj.ro (I.T.); laurian.vlase@umfcluj.ro (L.V.); 6 Department of Bromatology, Hygiene, Nutrition, Faculty of Pharmacy, Iuliu Hatieganu University of Medicine and Pharmacy, 6 Louis Pasteur, 400349 Cluj-Napoca, Romania; lfilip@umfcluj.ro; 7 Academy of Romanian Scientists (AOSR), 3 Ilfov Street, 050044 Bucharest, Romania

**Keywords:** *Juglans regia* L., walnut septum, diaphragma juglandis fructus, walnut pellicle, molecular docking, polyphenols, LC-QTOF-MS/MS, antioxidant, immunomodulatory, cell culture

## Abstract

**Background:** Walnut (*Juglans regia* L.) processing generates polyphenol-rich by-products, including the septum (WS) and pellicle (WP). This study comparatively characterized the phytochemical composition of WS and WP from Romania and investigated their antioxidant and preliminary immunomodulatory properties using chemical, computational, and cell-based approaches. **Methods:** Extracts were characterized by targeted LC–QTOF–MS/MS, and antioxidant capacity was evaluated using ABTS, DPPH, and FRAP assays. Major phenolic compounds were docked against Kelch-like ECH-associated protein 1 (KEAP1), followed by 100 ns molecular dynamics simulations. Cytocompatibility was evaluated in Caco-2, Jurkat, and THP-1 cells, and cellular antioxidant activity was assessed in H_2_O_2_-stimulated Caco-2 cells using the DCFH-DA assay. Immunomodulatory potential was investigated following lipopolysaccharide (LPS) stimulation of the same cell lines. **Results:** WSLE contained 25 quantified compounds, with ellagic acid, catechin, procyanidins, and quercitrin among the major quantified constituents, whereas WP contained 13, with higher ellagic acid, gallic acid, and castalagin levels. WPE showed greater cell-free antioxidant capacity, while both extracts attenuated the H_2_O_2_-induced increase in intracellular DCF fluorescence in Caco-2 cells. Simulations suggested favorable KEAP1 interactions, with quercitrin and catechin predicted to form the most stable complexes. LPS induced an insufficient cytokine response in Caco-2 and Jurkat cells. In THP-1 cells, IL-8 was the only responsive marker, and both extracts reduced its LPS-induced secretion. **Conclusions:** The extracts showed distinct polyphenolic profiles and redox-related cellular activity. Their effects on IL-8 secretion in THP-1 cells and predicted KEAP1 binding are preliminary and hypothesis-generating; Nrf2 activation was not measured, and validation in physiologically relevant *in vivo* and clinical models is required to establish the biological relevance and potential applications of these extracts.

## 1. Introduction

Oxidative stress and chronic low-grade inflammation are closely interrelated processes that contribute to the onset and progression of major non-communicable diseases, including cardiovascular disease, metabolic syndrome, type 2 diabetes, neurodegenerative disorders, and cancer [1,2,3,4]. Oxidative stress arises from an imbalance between the excessive production of reactive oxygen species (ROS) and reactive nitrogen species (RNS) and the endogenous antioxidant defense systems, resulting in oxidative damage to lipids, proteins, and DNA. This damage activates redox-sensitive signaling pathways mediated by nuclear factor kappa-light-chain-enhancer of activated B cells (NF-κB) and activator protein-1 (AP-1) [5,6], thereby contributing to the persistence of chronic inflammation and the disruption of cellular homeostasis [7,8]. In this context, dietary and pharmacological strategies involving natural bioactive compounds with antioxidant and anti-inflammatory properties are being investigated for disease prevention and as complementary therapeutic approaches [9,10,11].

Among tree nuts, walnuts (*Juglans regia* L.) have a particularly favorable nutritional and phytochemical profile. They contain various bioactive compounds, including polyunsaturated fatty acids (PUFAs), notably α-linolenic acid (ALA, 18:3 n-3), tocopherols, melatonin, and a wide range of polyphenolic compounds, such as phenolic acids, flavonols, flavan-3-ols, proanthocyanidins, and ellagitannins [12,13]. Clinical evidence, including meta-analyses of randomized trials, suggests that regular walnut consumption is associated with beneficial changes in some cardiometabolic health markers. Reported benefits include favorable changes in lipid profiles, reductions in certain inflammatory biomarkers, including C-reactive protein and interleukin (IL)-6, and improvements in endothelial function [14]. In addition to their antioxidant activity, walnut-derived bioactive compounds may support cardiovascular health by reducing intestinal cholesterol absorption, potentially through competition with dietary cholesterol for micellar incorporation [15,16,17].

The intestine represents a key site of action for these compounds. Increasing attention has focused on inflammatory bowel diseases and intestinal barrier dysfunction, which have been associated with the development and progression of several metabolic and autoimmune systemic diseases [18] as well as neuropsychiatric and neurodegenerative disorders [19]. Recent evidence has linked the gut–brain axis with aging and neurodegenerative diseases, emphasizing the relevance of modulating oxidative stress and inflammation at both intestinal and systemic levels [20]. Walnuts may also help maintain a healthy gut microbiome [21]. In addition, intestinal metabolism of walnut ellagitannins generates urolithins, microbial metabolites with antioxidant properties and reported local and systemic bioactivities, including anti-inflammatory, neuroprotective, and antitumor activities [22,23].

Although research has primarily focused on whole walnuts, recent attention has shifted toward extracts derived from their by-products. The walnut septum (diaphragma juglandis fructus) and the pellicle (seed coat) are generally discarded as low-value by-products during industrial processing [24,25]. Walnut septum (WS) contains high levels of phenolic acids, oligomeric proanthocyanidins (dimers and trimers), and derivatives of flavonols and flavan-3-ols, together with smaller quantities of ellagitannins [26,27,28,29]. In preclinical studies, WS extracts have demonstrated antioxidant, anti-inflammatory, antimicrobial, antidiabetic, antitussive, antiaging, and organ-protective effects, as well as cytotoxic and antitumor activities [27,28,29,30,31]. Walnut pellicle (WP) is a major source of walnut polyphenols. Its complex composition [32,33,34,35,36] varies according to genotype, environmental conditions, and post-harvest handling practices. The pellicle substantially contributes to the health-promoting properties of walnuts, as its removal has been reported to decrease the total antioxidant activity by approximately 90% [24]. This estimate may vary depending on the analytical method used. In addition to polyphenols, WP contains bioactive lipids, including oxylipins and endocannabinoid-related molecules [37]. WP extracts have shown strong antioxidant effects (inhibiting lipid peroxidation), antimicrobial and antifungal actions, and cytotoxic and antiproliferative activities against several cancer cell lines [38,39,40]. They may also modulate gut microbiota composition and metabolite profiles, largely through their ellagitannin content [41].

Despite these findings, several knowledge gaps remain. In previous studies, our group characterized WS from the Transylvania region by profiling its phenolic compounds, phytosterols [28], and tocopherols [29]. These studies reported inhibitory activity against acetylcholinesterase, tyrosinase, α-glucosidase, and lipase; antimicrobial activity; selective cytotoxicity toward A549, T47D, KBluc, and MCF-7 cells; and anti-inflammatory activity in human gingival fibroblasts [29]. However, the procyanidin and ellagic acid/ellagitannin contents of WS, as well as those of the pellicle derived from the same source, were not investigated. More broadly, WS and WP have predominantly been studied separately using different plant materials and extraction protocols, preventing reliable comparisons of their bioactivities. Their direct effects on intestinal epithelial and immune cells, which are central to intestinal barrier integrity and the gut–immune interface, also remain poorly characterized. Walnut by-products may represent a source of bioactive compounds for nutraceutical development. However, whether such preparations can protect intestinal barrier function or support the repair of a compromised intestinal barrier remains a hypothesis requiring direct experimental validation.

Accordingly, the present study aimed to compare the phytochemical composition and biological activities of WS and WP extracts obtained from the same walnut batch using an identical extraction protocol and experimental conditions. It was hypothesized that the two extracts would exhibit distinct phenolic profiles and, consequently, would differ in their antioxidant activities, as well as in their capacity to modulate cellular redox status and immune responses [41,42].

Therefore, the specific objectives of this study were: (i) to prepare polyphenol-rich extracts from walnut septum and walnut pellicle (hereafter referred to as WSE and WPE, respectively) harvested in Transylvania, Romania, and to characterize them phytochemically by determining their total phenolic, flavonoid, and condensed tannin contents, followed by targeted liquid chromatography–quadrupole time-of-flight tandem mass spectrometry (LC–QTOF–MS/MS) analysis for the quantification of individual polyphenols; (ii) to evaluate the antioxidant capacity of the extracts using the cell-free 2,2′-azino-bis(3-ethylbenzothiazoline-6-sulfonic acid) (ABTS), 2,2-diphenyl-1-picrylhydrazyl (DPPH), and ferric reducing antioxidant power (FRAP) assays; (iii) to investigate, through molecular docking and molecular dynamics simulations, the interaction of the main phenolic constituents identified by LC–QTOF–MS/MS with Kelch-like ECH-associated protein 1 (KEAP1); (iv) to assess the cytocompatibility and cellular antioxidant activity (AA) of the extracts in Caco-2 intestinal cells under basal and H_2_O_2_-induced oxidative stress conditions; and (v) to determine the cytocompatibility of the extracts and their ability to attenuate lipopolysaccharide (LPS)-induced pro-inflammatory responses in Caco-2 cells, as well as in THP-1 and Jurkat immune cell lines. Collectively, these objectives provide a basis for future investigations into the immunomodulatory potential of WSE and WPE, particularly at the gut–immune system interface.

To the best of our knowledge, this is the first study to compare septum and pellicle extracts from the same walnut batch using an identical extraction protocol, combining phytochemical profiling with cell-free antioxidant assays, computational analyses, and evaluation of biological effects in cultured cells.

## 2. Results and Discussion

WS and WP, by-products of walnut (*Juglans regia* L.) processing, are increasingly recognized as sustainable sources of bioactive polyphenols with potential relevance to human health. Although the pellicle is typically consumed with the kernel, it is often removed during industrial blanching, roasting, or the production of light-colored foods and subsequently discarded as waste [37]. Recovering both the pellicle and septum therefore represents a rational strategy for valorizing these residues as functional ingredients in the food, dietary supplement, pharmaceutical, and cosmetic sectors.

In the present study, extracts of WS and WP, obtained from the same walnuts harvested at maturity in Cluj-Napoca, Transylvania, Romania, were analyzed. To the best of our knowledge, this is the first detailed comparative investigation of the phytochemical composition of these Romanian walnut by-products, with particular emphasis on their polyphenolic profiles. Polyphenols are secondary metabolites of *Juglans regia* L. that may exert hormetic effects. Their biosynthesis is influenced by several factors, including cultivar, geographic origin, environmental conditions, agricultural practices, and post-harvest management. Accordingly, the phytochemical profiles described in this study should be interpreted in the context of the specific conditions under which the walnuts were cultivated, harvested, and stored.

Ultra-Turrax extraction (UTE), followed by vortex mixing, was selected for extract preparation. In a previous study by our group [28], optimal UTE conditions for the extraction of polyphenolic compounds from WS were established using an original experimental design. Compared with conventional maceration, UTE produced extracts with higher polyphenol contents and greater antioxidant activity [28]. The water/acetone system showed higher extraction efficiency for catechin, epicatechin, and quercetin glycosides, whereas water/ethanol was more effective for phenolic acids, including gallic, syringic, protocatechuic, and vanillic acids. Consistent with these observations, 80% acetone is widely recognized as an effective solvent for extracting condensed tannins [43]. The optimal conditions identified—water/acetone (50:50, *v*/*v*) as the extraction solvent, a solid-to-solvent ratio of 1:10 (*w*/*v*), 2 min of UTE, and a further 2 min of vortex agitation—were applied to both WS and WP matrices in the present study to obtain polyphenol-rich extracts. Because the aqueous-acetone system was selected to obtain a phytochemical profile with maximal antioxidant activity, the organic solvent was removed under reduced pressure before all biological assays, thereby reducing potential interference and cytotoxicity associated with the extraction solvent.

Vortex homogenization and Ultra-Turrax extraction are commonly used as sample preparation methods before other extraction procedures. However, they may also be applied directly for the extraction of bioactive compounds, particularly when producing extracts with high antioxidant activity [44,45]. By generating high shear forces, the Ultra-Turrax device promotes compound release from the plant matrix and enhances their interaction with the solvent [46]. This approach enables efficient extraction within a short time and with low energy consumption [47]. Compared with other green methods, including ultrasound- and high-pressure-assisted extraction, UTE is technically straightforward, requires limited capital investment, uses small solvent volumes, and presents a lower environmental risk, making it an economical and sustainable alternative [45].

Under the applied experimental conditions, extraction yields, calculated relative to the dry mass of the initial plant material, were 9.41% (*w*/*w*) for walnut septum and 24.27% (*w*/*w*) for walnut pellicle.

### 2.1. Total Phenolic, Flavonoid, and Condensed Tannin Contents of Crude Walnut Septum and Pellicle Extracts

Both extracts were characterized for total phenolic content (TPC), total flavonoid content (TFC), and condensed tannin content (CTC) (Table 1).

Both WSE and WPE contained substantial quantities of polyphenolic compounds; however, the value recorded for WPE was approximately 2.0-fold higher than that for WSE (Table 1). These results were evaluated against data reported in the scientific literature. Rusu et al. (2018) [28] reported lower TPC values (67.03 ± 9.76 mg gallic acid equivalents (GAE)/g dry weight (dw)) for WSE prepared under similar UTE conditions. Comparable values were obtained by Özşahin Delibaş and Kıray (2023) for WSE produced by Soxhlet extraction with 95% ethanol (119.42 ± 2.39 mg GAE/g dw) [31]. By contrast, Luksiene et al. (2025) reported higher values for WS extracts prepared by maceration in 50% ethanol (293.14 ± 0.07 mg GAE/g dw) or in 50% acetone (270.70 ± 3.53 mg GAE/g dw), ultrasonic-assisted extraction (UAE) in 50% ethanol (279.35 ± 5.44 mg GAE/g dw), or reflux in 50% acetone (291.45 ± 2.47 mg GAE/g dw) [48]. Markedly higher TPC values for WSE were also reported by Zhang et al. (2020) [32] following extraction with 70% methanol by condensation reflux (1111.26 ± 17.16 mg GAE/g dw), enzyme-assisted extraction (EAE) (834.93 ± 18.16 mg GAE/g dw), or UAE (938.85 ± 15.03 mg GAE/g dw). Variations among studies are largely attributable to differences in genotype, geographic origin, environmental conditions, extraction methodology, and solvent system [28,31,48].

Regarding WP, the total phenolic content determined in the extract is consistent with the phenolic levels previously reported for this matrix [49,50,51]. Trandafir et al. (2016) [49] and Akbari et al. (2012) [50] both obtained elevated TPC values (between 52.05 and 383.3 mg GAE/g dw) in WP across multiple genotypes, exceeding kernel values. Qingyang et al. (2023) [51] showed that TPC in WP is highly sensitive to post-harvest processing, particularly drying conditions. Overall, the literature consistently identifies WP as one of the most phenolic-rich walnut components, with TPC severalfold higher than in shells, green peel, kernels, and leaves [34,52]. These findings align with our results, which show substantially higher total phenolic content in WPE than in WSE (Table 1).

The TFC values of WSE and WPE in the present study were comparable and indicate that both matrices contain a moderate but biologically relevant flavonoid fraction (Table 1). The TFC value of our WSE is in close agreement with the maximum value reported by Rusu et al. (2018) for the extracts prepared under similar conditions (9.76 ± 0.23 mg quercetin equivalents (QE)/g dw) [28] and falls within the lower-to-mid range of flavonoid levels observed in other walnut tissues, such as leaves [53]. Previous reports on WS showed that flavonoids, mostly proanthocyanidins, generally represent a minor fraction of total phenolics [28]. For our WPE, the TFC clearly exceeds the low flavonoid values (between 0.18 and 0.69 mg QE/g) reported by Trandafir et al. (2016) in extracts obtained from the same matrix [49], likely reflecting differences in genotype, ripeness, and pellicle color, as well as extraction conditions [35,49,54]. UHPLC-MS/MS analyses performed by Wang et al. (2022) [35] further support an abundance of flavonoids in dark-colored pellicles, especially in the form of flavanols such as (+)-catechin and epicatechin.

Regarding CTC, both extracts showed substantial levels of condensed tannins, although the WSE had a 2.9-fold higher content than the WPE (Table 1). However, the absolute values should be interpreted with caution, since the vanillin–HCl assay is a colorimetric method with limited specificity for proanthocyanidins [28]. This method is frequently used to determine levels of condensed tannins (or proanthocyanidins, oligomeric and polymeric forms of flavan-3-ols). It is preferred because it is specific to a narrow range of flavanols and dihydrochalcones that have a single bond at the 2,3-position and free hydroxyl groups oriented in the meta position on ring B [55]. However, catechin and condensed tannins react differently with vanillin [55], depending on time and the degree of polymerization of the condensed tannin [56]. This can lead to underestimation or overestimation of condensed tannin concentration, especially in matrices with high tannin content [57]. Using catechin equivalents (CE) often overestimates tannin content [55]. For this method, there are also other well-documented variables, dependent on testing conditions, that require careful interpretation of the results: the response varies significantly depending on the substitution of ring A and the stereochemistry of the flavan-3-ols; interfering substances (sugars, anthocyanins, aldehydes, and ascorbate) can enhance or suppress color development; and the results depend largely on the calibration standard used [58]. Furthermore, tannins are generally extracted using aqueous acetone; it is more effective than other organic solvents, such as methanol and ethanol, for extracting them from plant matrices [28,59,60]. However, acetone reacts with vanillin in an acidic medium, forming a chromogen with a maximum at 548 nm, which introduces additional error in determining condensed tannins [59]. Although these methodological limitations are acknowledged, parallel analysis under identical conditions supports comparison, although matrix-dependent assay responses may still affect the results. For the reasons outlined above, absolute CTC values expressed in mg CE/g dw cannot be interpreted as the actual mass percentage of condensed tannins.

Nevertheless, the CTC values of our WSE align with those previously reported for the extracts prepared by UTE under the same conditions (237.20 ± 3.22 mg CE/g dw) [28] and are significantly higher than those reported for the extracts prepared by other classical or alternative extraction techniques [32]. Thus, Zhang et al. (2020) reported lower CTC values for WS extracts prepared using 70% methanol and three extraction methods: 42.65 ± 1.58 mg CE/g dw by methanolic condensation reflux extraction (ME), 33.92 ± 0.12 mg CE/g dw by UAE, and 45.29 ± 0.88 mg CE/g dw by EAE [32]. In contrast, the same authors reported markedly higher levels of CTC in walnut pellicle: 573.19 ± 21.54 mg CE/g dw by ME, 727.32 ± 19.21 mg CE/g dw by UAE, and 699.78 ± 22.66 mg CE/g dw by EAE [32]. This finding underscores the substantial impact of analytical method, extraction method, solvent system, and walnut origin on tannin recovery [32].

In our study, WSE contained approximately three times as much condensed tannins, expressed as catechin equivalents per unit dry plant material, as WPE. However, the existing literature typically identifies the pellicle as the main reservoir of tannins [32,33,40]. The phytochemical analysis via LC–MS/MS (presented in Section 2.3) revealed that the septum is a concentrated source of condensed tannins, with higher levels of catechin and proanthocyanidins, while the pellicle contains primarily ellagitannins, with higher levels of ellagic acid and castalagin.

### 2.2. Antioxidant Activities of Crude Extracts

The crude WS and WP extracts were characterized by quantifying their total phenolic, flavonoid, and condensed tannin contents. Antioxidant activity was initially assessed using abiotic assays to determine the antioxidant potential of the extracts and establish whether subsequent investigation in cell culture models was justified. Table 2 compares the antioxidant activities of WSE and WPE.

WPE showed significantly higher antioxidant capacity than WSE in all three abiotic assays: ABTS, DPPH, and FRAP (*p* < 0.05) (Table 2).

The ABTS assay, widely applied for comparative evaluation of total antioxidant activity in complex plant extracts [61,62,63], revealed an ABTS•^+^ radical scavenging activity approximately 2.0 times higher in WPE than in WSE (*p* < 0.05) (Table 2). Compared with previously reported data, the AA-ABTS values obtained for our WSE moderately exceeded those reported by Rusu et al. (2018) [28] for WS extracts prepared by UTE using 50% or 75% acetone (168.62 and 174.28 mg Trolox equivalents (TE)/g dw, respectively), suggesting that differences in raw material origin and harvest year may affect the measured antioxidant capacity. The higher AA-ABTS values observed for WPE correlate with its greater TPC value and with the recognized contribution of phenolic acids and flavonoids in the pellicle to free radical scavenging and metal chelation [64,65,66].

The DPPH assay indicated approximately 2.3 times stronger free radical-scavenging activity in WPE than in WSE (*p* < 0.05) (Table 2), suggesting a higher accumulation of hydrogen/electron-donating constituents in the pellicle [63,67]. The AA-DPPH of our WSE was comparable to that reported by Rusu et al. (2018) for septum extracts obtained under identical UTE conditions (255.89 mg TE/g dw) [28], confirming the robust antioxidant potential of WS. Literature values for related nut matrices, including walnuts [24] and pecan kernels and shells [68], as well as higher DPPH decolorization in hazelnut kernels and pellicles versus oil [69], further emphasize the importance of phenolic-rich tissues—particularly the pellicle—as key antioxidant reservoirs.

The results of the FRAP assay indicated a ferric reducing antioxidant power approximately 1.4 times higher for WPE compared to WSE (*p* < 0.05) (Table 2), suggesting a greater electron-donating capacity and elevated levels of redox-active compounds within the pellicle [63]. Notably, the AA-FRAP value for our WSE exceeded the value reported by Rusu et al. (2018) for the septum extract obtained using the same UTE protocol (400.97 mg TE/g dw) [28], but it remained lower than that of WPE. This observation further emphasizes the pellicle matrix’s superior reducing capacity.

The higher total phenolic content observed in WPE (Table 1) was consistent with its higher ABTS, DPPH, and FRAP values (Table 2), indicating that its phenolic composition likely contributed to the stronger antioxidant capacity measured under the experimental conditions. Previous research has indicated that the distribution of phenolic compounds varies specifically between kernels and pellicles [54,70], with pellicles generally exhibiting higher TPC and superior antioxidant profiles. Furthermore, these distributions are influenced by factors such as the stage of maturation and environmental conditions [54].. Previous studies have also discussed the potential relationships among walnut-derived phenolic compounds, antioxidant effects, and oxidative-stress-related biological responses [61,66,70,71,72]. Consistent with these observations, our results indicated antioxidant activity in walnut septum and pellicle extracts under the experimental conditions used, with WPE showing particularly high activity. These findings warrant further investigation into the biological effects of these extracts.

### 2.3. Analysis of Individual Polyphenols by LC-QTOF-MS/MS

Lyophilized extracts were selected for the planned cell-based studies to minimize potential cytotoxicity associated with the extraction solvent and its interference with the biological responses under investigation. Because lyophilization may induce physicochemical changes in some bioactive compounds, individual phenolic compounds were analyzed in the lyophilized extracts (WSLE and WPLE, respectively) rather than in the corresponding crude extracts. This approach provides compositional information on the preparations intended for use in cell-based assays, offering a relevant basis for investigating potential relationships between phenolic composition and biological activity.

Detailed phytochemical analysis of the lyophilized extracts, performed using a recently developed LC-QTOF-MS/MS method (Table S1), revealed slightly different phenolic profiles, both qualitatively and quantitatively, between the two matrices studied (Table 3). These findings highlight the phytochemical diversity of the walnut by-products investigated and support further research into the potential contributions of their phenolic constituents to biological activity.

WSLE exhibited a complex and diverse polyphenolic profile, whereas WPLE showed a more concentrated profile, quantitatively dominated by ellagic acid (EA), gallic acid (GA), catechin, procyanidins, and castalagin. The extracts contained phenolic acids, flavonoids, and phenylpropanoids, while castalagin was quantified only in WPLE The WSLE was characterized by high ellagic acid (derived from ellagitannin hydrolysis), abundant catechin/procyanidin derivatives, quercitrin, and, notably, the absence of the analyzed ellagitannins, castalagin and vescalagin. Other hydroxycinnamic acids, flavones, coumarins, and phenylpropanoid aldehydes were detected at lower concentrations, mostly in the walnut septum.

Gallic acid and other hydroxybenzoic acids, together with ellagic acid, were important phenolic constituents of the extracts. EA was present in both extracts but was clearly more abundant in WPLE (8377.06 µg/g) than in WSLE (5228.94 µg/g) (Table 3), confirming the pellicle as an important EA reservoir consistent with earlier studies [32,33,36,41,73]. When comparing the values obtained, expressed in µg/g dw of plant material, the EA content of our extract derived from WS (492.14 μg/g dw) is comparable to that reported in previous studies (239.65–1733.64 µg/g) for extracts in 50% or 70% methanol, obtained using various extraction methods, from samples collected in different geographic regions [26,32]. In contrast, the WP analyzed in this study had a much higher EA content compared to that reported for an extract prepared in 80% methanol (354.46–423.32 µg/g) [73]. The abundance of EA in WPLE (8377 µg/g dw)—which substantially exceeds that in WSLE (5229 µg/g dw)—could be associated with WPE’s superior antioxidant capacity in the ABTS assay (428.14 mg TE/g dw versus 210.40 mg TE/g dw) and may contribute to its AA-FRAP activity (916.36 mg TE/g dw).

GA was also detected in both matrices, with higher levels in WPLE (706.01 µg/g). Its content in WS (40.60 µg/g dw) was below the ranges previously reported for walnut septum obtained under different extraction conditions (79.58–272.52 µg/g) [26,29,74] which may be attributed to differences in extraction parameters, plant origin, or sample pretreatment. The higher concentration of GA in WPLE compared to WSLE is consistent with, and could partially account for, the greater radical-scavenging activity observed [75].

Other hydroxybenzoic acids (protocatechuic, syringic, and vanillic acids) showed a pronounced distribution in the septum, being abundant in WSLE and low or undetectable in WPLE. This pattern is consistent with literature indicating that these compounds are sensitive to walnut maturity and decline in late-harvest pellicles [26,29,32,54].

Hydroxycinnamic acids further differentiated the two matrices. The WSLE exhibited a broader hydroxycinnamic profile (e.g., caffeic acid, 4-*O*-caffeoylquinic acid, chlorogenic acid, and *p*-coumaric acid), whereas the WPLE contained only low amounts of chlorogenic and *p*-coumaric acids, while caffeic acid remained below the limit of quantification (Table 3). The measured concentrations generally fall within or below the ranges reported for walnut septum and pellicle in previous work [26,32,36,73,74], suggesting that hydroxycinnamic acids contribute substantially to the phenolic profile of the WSLE. Their strong intrinsic antioxidant properties [76,77] indicate that they may make a significant contribution to the overall redox activity of both extracts.

Flavan-3-ols and procyanidins constitute a dominant flavonoid subclass. The WSLE was particularly rich in catechin, epicatechin, gallocatechin, and several procyanidin oligomers (B1, B3, B4), whereas the WPLE contained catechin and procyanidins at lower levels and lacked detectable epicatechin and gallocatechin (Table 3). The catechin content of the WSLE (3989.51 µg/g) is broadly in line with the upper ranges reported for walnut septum (589–9989.16 µg/g) [26,29,32,74,78], while the pellicle catechin level in WP (351.38 µg/g dw WP) exceeds the value previously reported (40.7 µg/g) for a 70% methanolic extract [70]. With regard to proanthocyanidins, Luksiene et al. (2025) determined the total proanthocyanidin content spectrophotometrically in WS samples from various regions of Lithuania, Ukraine, and Armenia and obtained significant values ranging from 1.14 to 7.65 mg epicatechin equivalents/g dw [48]. The total procyanidin content measured in our WSLE by high-resolution LC-MS (LC-HRMS) was 5.10 mg/g (0.48 mg/g expressed per g dw WS) (Table 3). The high content of flavan-3-ols in WSLE—catechin (3990 µg/g dw), procyanidin B1 (2082.28 µg/g dw), and procyanidin B3 (2786 µg/g dw) (Table 3)—could indicate that these compounds are also abundant in crude extracts and could therefore contribute to the strong antioxidant activities obtained in the ABTS (210.40 mg TE/g dw) and DPPH (235.94 mg TE/g dw) assays for WSE (Table 2), despite TPC values being lower than those of WPE (Table 1). These data support the septum as a particularly flavan-3-ol-rich matrix and suggest that proanthocyanidins may contribute substantially to its antioxidant capacity, although this relationship remains indicative, as discussed in the Limitations section.

The flavonoid profile showed distinct enrichment of flavonols and flavones in the septum. The WSLE contained appreciable levels of quercetin, quercitrin, isoquercitrin, hyperoside, and apigenin. In contrast, the WPLE showed only modest amounts of hyperoside and isoquercitrin, with the other flavonols and flavones below the limit of detection (Table 3). The concentrations observed for these compounds in the WSLE fall within or above the ranges reported for walnut septum in previous LC-based studies [26,29,32,74], and exceed those documented for pellicle [54]. Thus, quercitrin, the most abundant flavonol, showed a higher level (3112.64 µg/g) than those reported by Rusu et al. (2020) for a lyophilized extract prepared with 50% acetone (1073.04 µg/g) [29], while Chen et al. (2022) reported 6816.18 µg/g for a lyophilized extract prepared in 80% ethanol using UAE [74]. This confirms that flavonols and flavones are associated with septum tissue and supports their proposed contribution to the broader spectrum of biological activities attributed to the WSLE.

In addition, several coumarins (esculetin, scopoletin, and umbelliferone) and phenylpropanoid aldehydes (coniferyl aldehyde and sinapaldehyde) were detected exclusively in the WSLE (Table 3). Their absence in the WPLE contrasts with review-based assumptions that tend to associate coumarins primarily with pellicles [36,40] and highlights the importance of direct analytical evidence in defining matrix-specific phytochemical distributions.

Ellagitannins (ETs) emerged as a key distinguishing feature between the two extracts. Castalagin was exclusively identified and quantified in the WPLE and was absent from the WSLE (Table 3), consistent with earlier reports of castalagin/vescalagin isomers and ellagic acid derivatives in walnut pellicles [32,33,73,79]. It is worth noting the high castalagin content in our WPLE (591.81 µg/g or 143.63 µg/g expressed per g dw WP) compared to the range reported by Medic et al. (2021) of 11.7–30.06 µg/g for the castalagin/vescalagin isomers [33]. The coexistence of substantial castalagin and EA levels in the WPLE indicates a prominent ellagitannin pool in the pellicle and reinforces its potential for robust antioxidant and anti-inflammatory effects. Given that ETs and EA serve as precursors of urolithins—bioactive metabolites produced by the gut microbiota and implicated in improved lipid metabolism, adipocyte browning, antitumor and anti-inflammatory activities, and neuroprotection [40,80]—the pellicle-specific abundance of castalagin holds significant functional relevance. As a hydrolysable tannin, castalagin exhibits an intrinsically stronger ferric reducing capacity than procyanidins and catechins that predominate in WS [32,33,73,79]. This could partly explain the 1.4-fold higher antioxidant activity of WPE compared to WSE, as observed in the FRAP assay (Table 2).

These matrix-specific phytochemical profiles, as described above, are consistent with the observed differences in the antioxidant behavior and biological activities of WS and WP and support their targeted valorization in functional, nutraceutical, and pharmaceutical applications. The identified polyphenolic compounds, together with their quantified levels in the extracts, may contribute to the observed biological effects and provide a basis for future dose–response investigations. However, these compounds should be regarded as likely contributors to overall antioxidant capacity rather than its sole determinants, as discussed further in the Limitations section.

### 2.4. Molecular Docking and Molecular Dynamics Simulations

For the main polyphenolic constituents quantified by LC-HRMS (Table 3), in silico molecular docking analyses and molecular dynamics simulations were performed to investigate their potential binding interactions with the nuclear factor erythroid 2-related factor 2 (Nrf2)-binding pocket of the KEAP1 protein. KEAP1 was selected as a molecular target in these in silico studies due to its critical role as the primary regulator of the KEAP1–Nrf2/Antioxidant Response Element (ARE) cellular signaling pathway. KEAP1 functions as a sensor. In the cytoplasm, it forms a complex with Nrf2, inactivating it and directing it toward proteasomal degradation. In the presence of electrophilic molecules, such as ROS, various polyphenols, etc., KEAP1 changes its conformation, releasing Nrf2, which can then be phosphorylated and translocated into the nucleus, where it acts as a transcription factor, directly or indirectly regulating the expression of over 2000 genes involved in the antioxidant defense system, detoxification, and the regulation of cytoprotection and proteostasis mechanisms [81]. Therefore, polyphenol binding to KEAP1 is the first step in activating this signaling pathway, which is essential for cellular redox regulation.

#### 2.4.1. Molecular Docking Results

Table 4 presents the binding affinity of ellagic acid, gallic acid, catechin, procyanidin B1, procyanidin B3, and quercitrin to KEAP1 from the molecular docking study.

**Figure 1:**
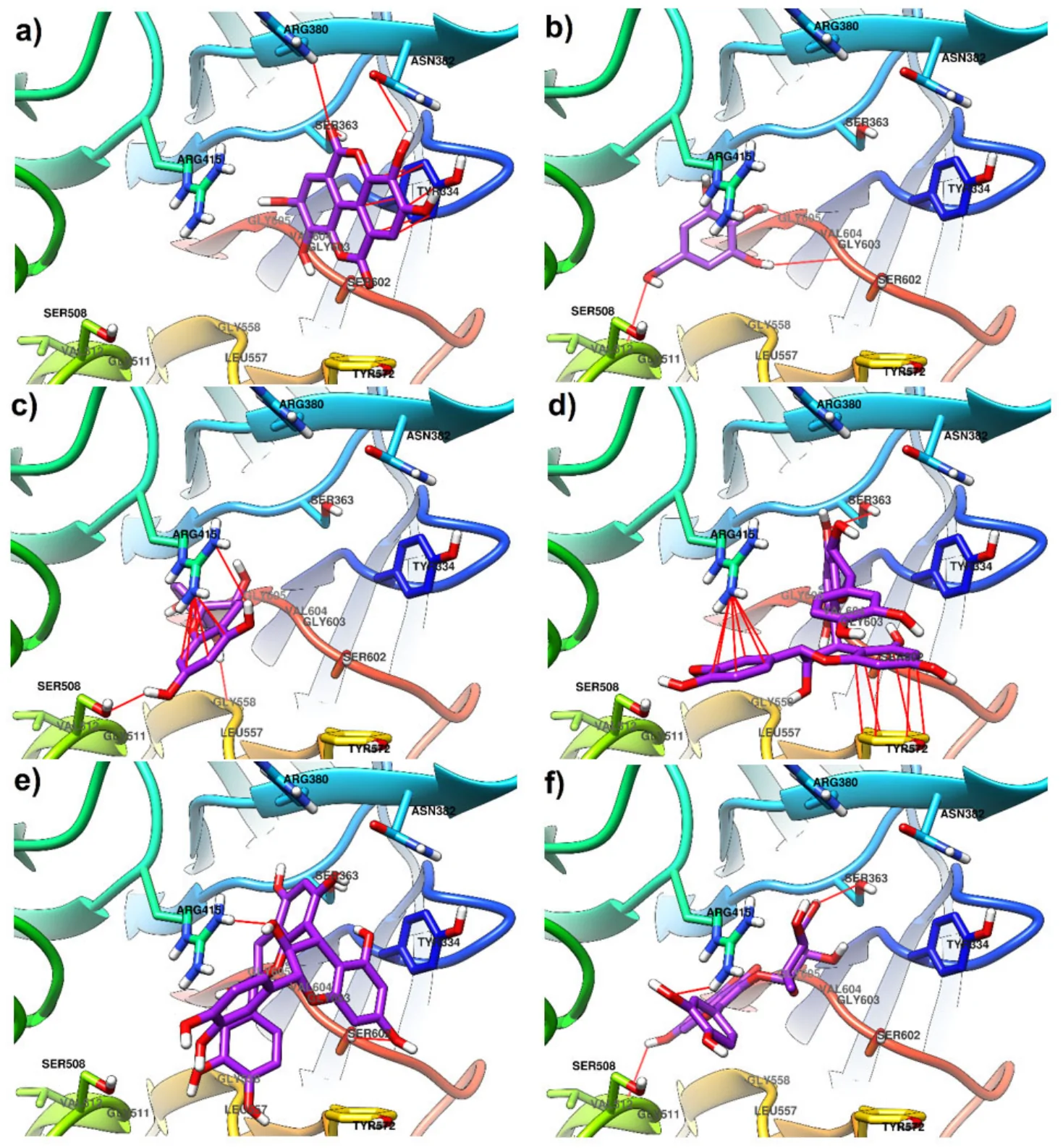
The predicted binding poses of ellagic acid (**a**), gallic acid (**b**), catechin (**c**), procyanidin B1 (**d**), procyanidin B3 (**e**), and quercitrin (**f**) to KEAP1.

The compound with the highest predicted affinity for KEAP1 was quercitrin (ΔG = −9.4 kcal/mol), followed by procyanidin B1 (ΔG = −8.7 kcal/mol), ellagic acid (ΔG = −8.4 kcal/mol), procyanidin B3 (ΔG = −8.0 kcal/mol), catechin (ΔG = −7.9 kcal/mol), and gallic acid, which showed the lowest affinity (ΔG = −6.3 kcal/mol) (Table 4). Figure 1 shows the graphical depictions of the binding poses of the studied compounds.

A relationship between molecular size and binding location was observed among the studied compounds. Smaller molecules, including gallic acid and catechin, were positioned deeper within the binding pocket, in a region delimited by Gly558, Gly605, Gly511, and Arg415. By contrast, larger compounds were located closer to the pocket entrance (Figure 1).

For ellagic acid, the exocyclic oxygen atoms of the lactone groups were predicted to function as hydrogen-bond acceptors for Ser363 and Ser602 and to participate in a polar (charge–dipole) interaction with the positively charged side chain of Arg380. In addition, a phenolic hydroxyl group was predicted to donate a hydrogen bond to the amide oxygen of the Asn382 side chain, whereas the catechol ring was predicted to establish a π–π stacking interaction with Tyr334. For gallic acid, the carboxylic group was predicted to form a hydrogen bond with the Gly511-Val512 peptide bridge, while two phenolic hydroxyl groups were predicted to interact with the Gly603-Val604-Gly605 peptide bridge (Figure 1).

For catechin, the dihydroxybenzene moiety attached to the chroman scaffold was predicted to interact with the Arg415 side chain through π-cation and hydrogen-bond interactions. The phenolic group at position 7 of the chroman was predicted to donate a hydrogen bond to the Ser508 side chain, whereas the phenolic group of the dihydroxybenzene moiety attached to position 2 was predicted to donate a hydrogen bond to the Leu557-Gly558 peptide bridge. The benzene rings of procyanidin B1 were predicted to engage in π–π stacking with Tyr572 and in a π-cation interaction with the positively charged Arg416 side chain. Simultaneously, the phenolic group at position 5 of the chroman was predicted to act as a hydrogen-bond acceptor for the Ser363 side chain (Figure 1).

In contrast, procyanidin B3 was predicted to bind to the KEAP1 site predominantly through van der Waals interactions, with only one polar interaction identified. Specifically, the hydroxyl group at position 3 of the chroman was predicted to act as a hydrogen-bond acceptor for the Arg415 side chain. Quercitrin was predicted to donate hydrogen bonds from the phenolic group at position 7 of 4H-chromen-4-one to the Gly511-Val512 peptide bridge and from a rhamnose hydroxyl group to the Ser363 side chain. Additionally, a phenolic group within the dihydroxybenzene moiety attached at position 2 of 4H-chromen-4-one was predicted to act as a hydrogen-bond acceptor for Arg415 (Figure 1).

#### 2.4.2. Molecular Dynamics Results

**Figure 2:**
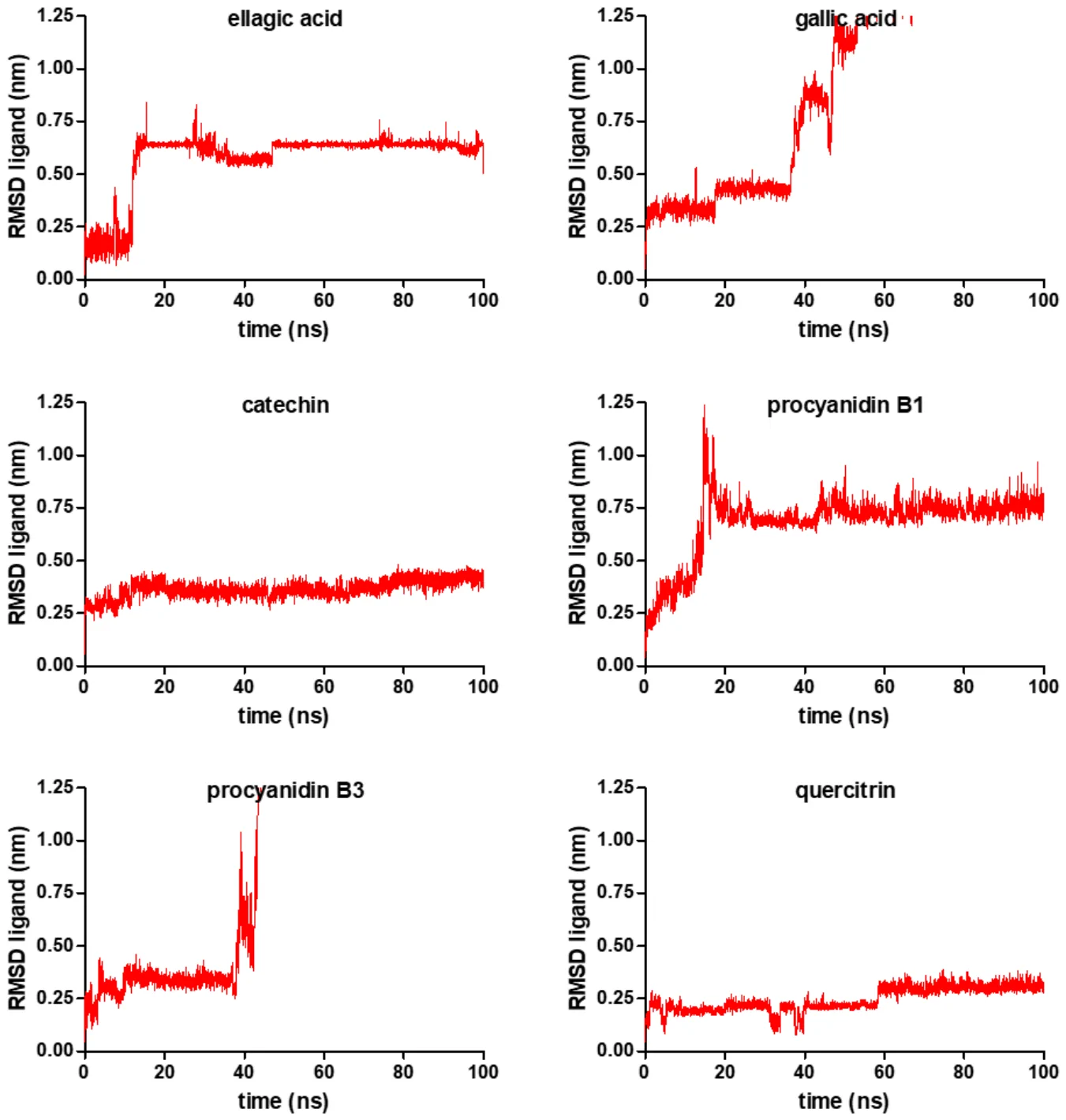
The RMSD of the coordinates of the heavy atoms of ellagic acid, gallic acid, catechin, procyanidin B1, procyanidin B3, and quercitrin bound to KEAP1.

Although docking predicted favorable binding poses for all investigated compounds, molecular dynamics (MD) simulations were subsequently performed to evaluate the stability of these interactions under dynamic physiological conditions. MD simulations revealed marked differences in the ability of the investigated compounds to remain stably bound within the KEAP1 binding pocket. Table 5 summarizes the quantitative analysis of the trajectories, while Figure 2 presents the evolution of ligand root-mean-square deviation (RMSD) over time.

**Figure 3:**
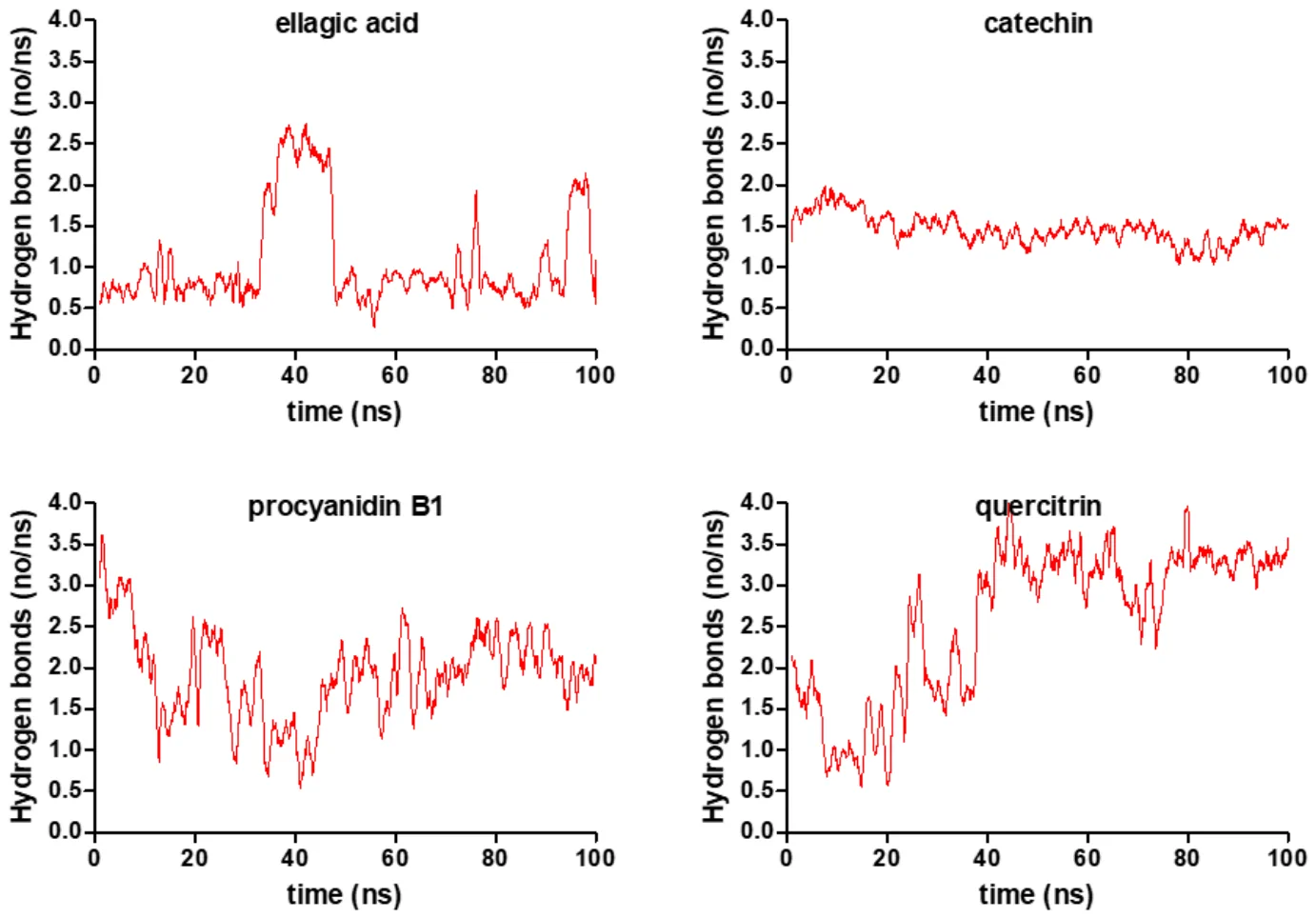
The hydrogen bonds between ellagic acid, catechin, procyanidin B1, and quercitrin and KEAP1 (1 ns moving average).

Quercitrin and catechin exhibited the highest stability, maintaining low RMSD values of 0.24 ± 0.05 nm and 0.36 ± 0.04 nm, respectively, with only minor fluctuations throughout the 100 ns simulations (Table 5). Ellagic acid displayed intermediate stability (0.57 ± 0.15 nm), undergoing a reorientation within the binding pocket during the simulation. In contrast, procyanidin B1 showed increased mobility within the binding pocket, reflected in a higher RMSD (0.68 ± 0.14 nm) and more pronounced fluctuations due to reorientation during the simulation (Table 5). By comparison, gallic acid and procyanidin B3 showed a progressive increase in ligand RMSD, reaching approximately 1.2–1.3 nm, consistent with their dissociation from the binding pocket during the simulations. Overall, the RMSD trajectories clearly distinguished two stable complexes (quercitrin and catechin), two complexes with moderate to low stability (ellagic acid and procyanidin B1), and two unstable complexes (gallic acid and procyanidin B3) that failed to remain bound under dynamic conditions and were discarded from further analysis (Table 5, Figure 2). In general, ligands that remained anchored in the binding site formed, on average, more hydrogen bonds than those that dissociated (Figure 3).

Quercitrin, the most stable ligand, also exhibited the highest average number of hydrogen bonds (2.61 ± 1.25), suggesting that an extensive network of polar interactions contributes to its retention in the binding cavity. Catechin follows the same trend, having both a low RMSD (0.36 ± 0.04 nm) and a relatively high number of hydrogen bonds (1.46 ± 0.66). Interestingly, procyanidin B1 forms a higher average number of hydrogen bonds (1.92 ± 0.97) than catechin and ellagic acid but showed lower complex stability (Table 5, Figure 3). This result suggests that complex stability depends not only on the number of hydrogen bonds but also on their persistence, hydrophobic, π–π, and van der Waals interactions, and the ligand’s orientation in the binding pocket. Thus, a higher number of hydrogen bonds does not necessarily guarantee higher stability if these interactions are transient or are compensated by increased ligand mobility, as observed for procyanidin B1.

The presence of stable ligands had only a minimal influence on the overall structure of KEAP1. The backbone RMSD values remained within a narrow range (0.14–0.18 nm), comparable to that of the apo protein (0.17 ± 0.04 nm), indicating that ligand binding did not induce significant conformational changes (Table 5). Likewise, the root mean square fluctuation (RMSF) values of both αC atoms and side chains were very similar among all stable complexes and the apo form, suggesting that residue flexibility was largely preserved rather than amplified and that ligand binding did not perturb the native dynamics of KEAP1 (Table 5). The radius of gyration (Rg) also remained essentially constant (1.80–1.81 nm), showing that KEAP1’s global compactness was maintained throughout the simulations regardless of the bound ligand (Table 5).

**Figure 4:**
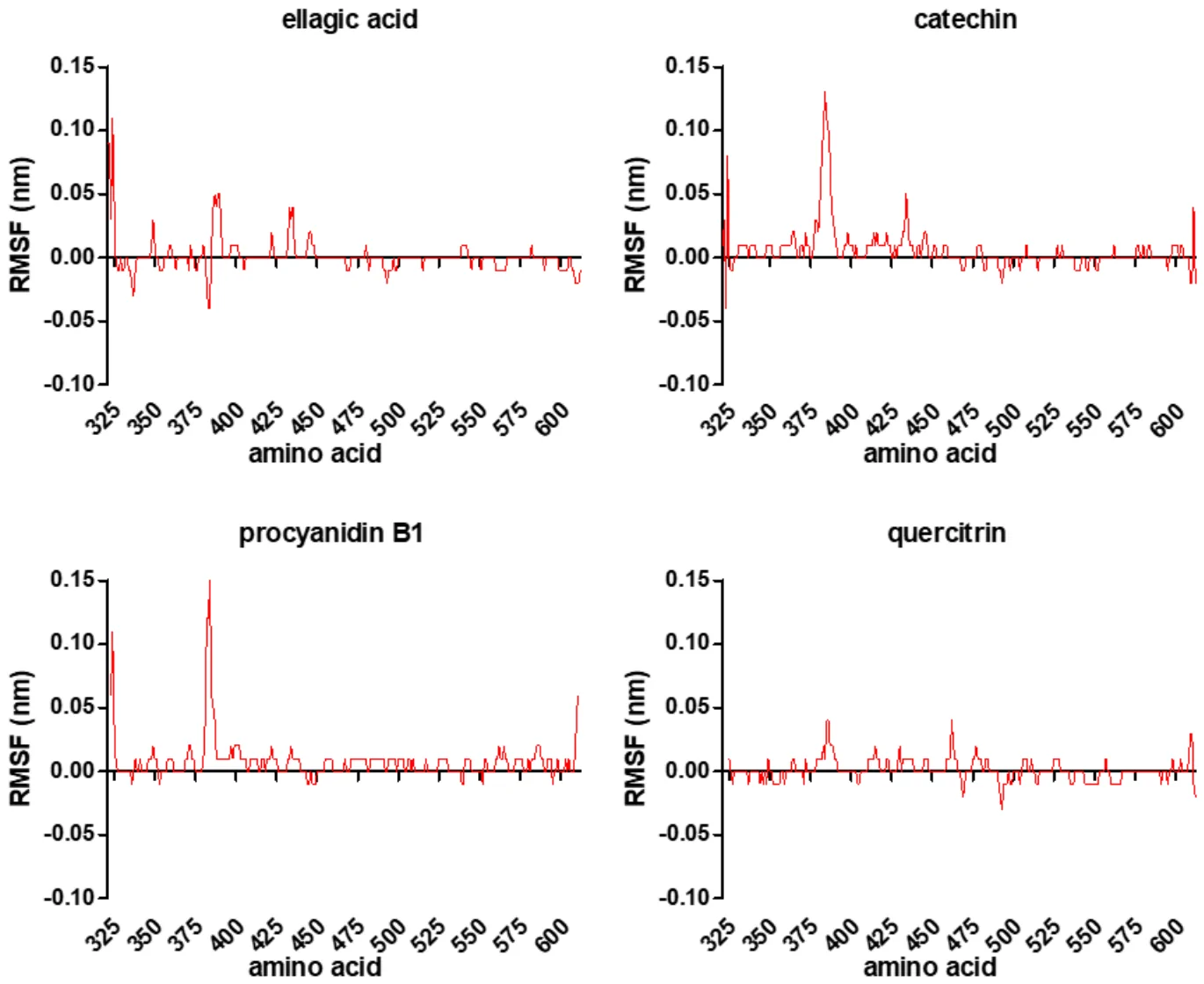
Difference in RMSF of amino acid αC atom coordinates, compared to the apo protein.

Analysis of interactions between ellagic acid, catechin, procyanidin B1, quercitrin, and KEAP1 at the amino acid level agrees with our previous observations. The RMSF analysis showed that ligand binding induced only localized changes in KEAP1 flexibility, while the protein’s overall dynamics remained comparable to the apo structure (Figure 4).

For the ellagic acid and quercitrin complexes, differences in αC atom fluctuations relative to the apo protein were generally small, with most residues showing deviations below ±0.05 nm. In contrast, complexes with catechin and procyanidin B1 showed higher fluctuations. RMSF values exceeded 0.1 nm for amino acids near the bound ligands catechin and procyanidin B1, particularly between Asn382 and Gly378. Several other residues near the binding site showed slightly increased flexibility. Conversely, other regions became marginally less flexible, indicating a redistribution rather than an overall increase in protein mobility (Figure 4).

**Figure 5:**
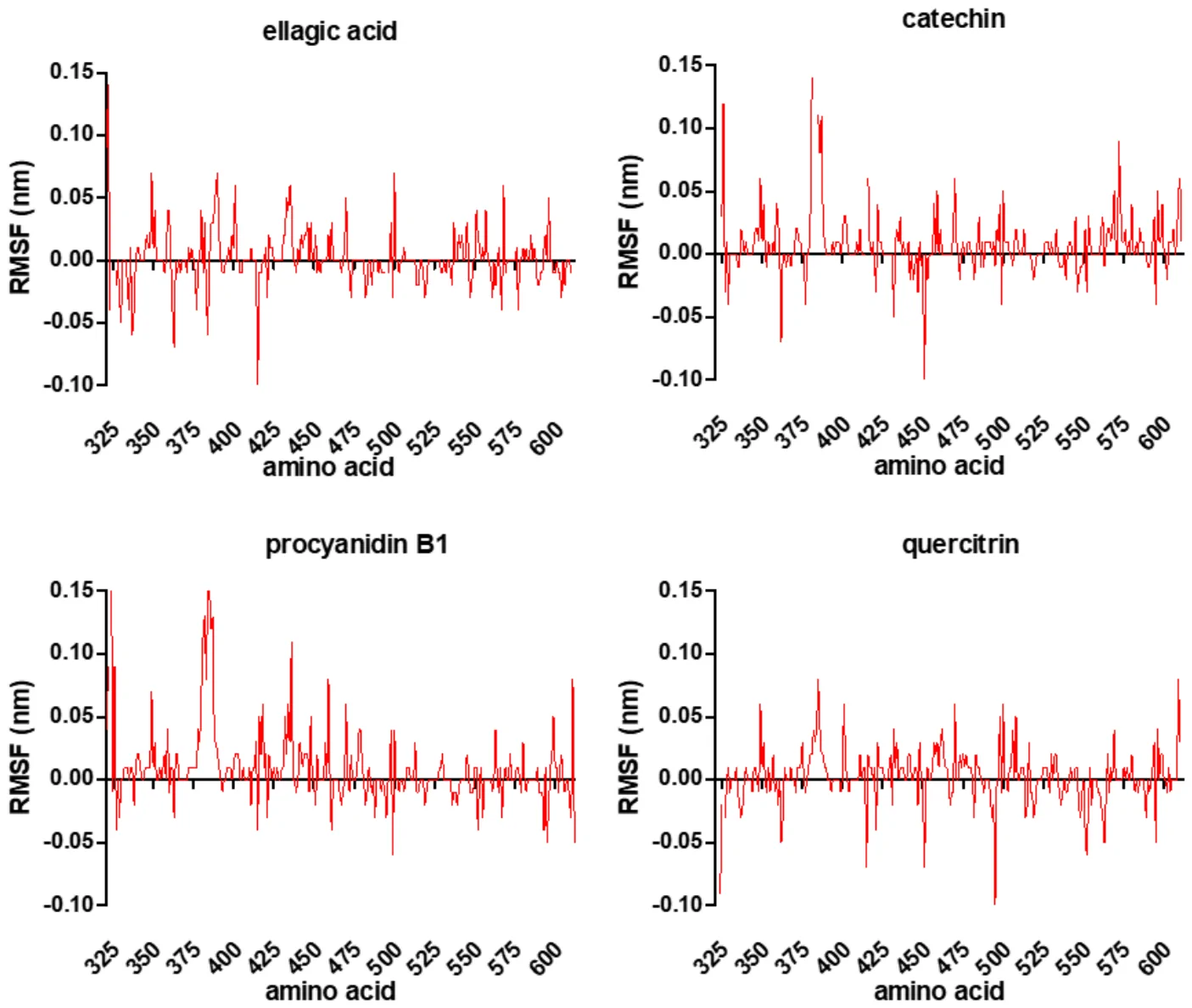
Difference in RMSF of amino acid side-chain atom coordinates, compared to the apo protein.

A similar trend was observed in the side-chain RMSF profiles (Figure 5). Although the fluctuations were generally higher than those of the backbone, the data reflect the greater conformational freedom of amino acid side chains.

Quercitrin and catechin produced only minor perturbations in residue flexibility, consistent with their stable binding modes observed during the RMSD analysis. Catechin and procyanidin B1 induced more pronounced local fluctuations, consistent with their higher ligand mobility throughout the simulations. Nevertheless, none of the stable ligands caused extensive conformational rearrangements in KEAP1, consistent with the low backbone RMSD (0.14–0.18 nm) and the nearly constant radius of gyration (1.80–1.81 nm), indicating that the overall protein fold remained structurally preserved during the 100 ns simulations. Unlike the backbone RMSF profiles, which showed only minor deviations from the apo structure, the sidechain RMSF profiles revealed both positive and negative fluctuations. The negative ΔRMSF values indicate that ligand binding reduced the mobility of specific amino acid side chains, whereas positive values reflect localized increases in flexibility (Figure 5). These observations suggest that ligand recognition is accompanied by a redistribution of local residue dynamics rather than an overall increase in protein flexibility, without affecting KEAP1’s global dynamics.

Our findings agree with previous computational studies reporting favorable KEAP1 binding for ellagic acid and catechin. However, extending the simulations to 100 ns revealed differences in the long-term stability of the complexes that could not be appreciated in previous 10 ns simulations [82,83]. Gallic acid showed the lowest docking affinity for KEAP1 and, in the molecular dynamics simulations, dissociated from the binding pocket; the resulting complex was therefore excluded from further trajectory analysis [82,84,85].

Both investigated extracts were rich in ellagic acid, catechin and procyanidins, whereas quercitrin was abundant in WSE. Given the previously reported links between these phenolic compounds and Nrf2-related signaling, the phenolic profiles of WSE and WPE provide a rationale for investigating the Nrf2 pathway in future studies. These in silico findings are hypothesis-generating and do not establish a mechanism for the cellular antioxidant activity of the extracts, as Nrf2 activation was not measured in this study. This hypothesis should be tested by assessing Nrf2 phosphorylation, nuclear translocation, or the expression of downstream target genes. Given the role of Nrf2 signaling in redox homeostasis [85], such studies would help clarify whether walnut septum and pellicle extracts influence cellular redox balance; any potential health relevance would require confirmation in appropriate in vivo and clinical studies.

### 2.5. Biological Activities of the Walnut Septum and Pellicle Lyophilized Extracts

The lyophilized extracts (WSLE and WPLE) were used in cell line studies for several reasons: (a) lyophilization removes interference from the extraction solvent, which may exhibit cytotoxic effects or otherwise impact results in cellular systems; (b) lyophilization improves the long-term stability of bioactive compounds, as cell-based assays are frequently conducted over extended periods; and (c) bioactive compounds from lyophilized extracts can be completely solubilized in a small volume of dimethyl sulfoxide (DMSO). Subsequently, dilutions were made directly in the culture medium to formulate working solutions at various concentrations, thus eliminating the influence of the extraction solvent and ensuring precise assessment.

To assess the biological activities of the WSLE and WPLE, cytocompatibility tests were conducted using three cell lines: Caco-2 (a human intestinal cell line), Jurkat (a human T lymphocyte cell line widely used in immunology research), and THP-1 (a human monocytic cell line used to study inflammatory responses). Furthermore, the antioxidant potential of the extracts was evaluated in Caco-2 cells, whereas their anti-inflammatory activity was assessed in Caco-2, Jurkat, and THP-1 cells.

The Caco-2 cell line is a well-established, physiologically relevant in vitro model for investigating the intestinal absorption and bioactivity of polyphenolic compounds. Upon differentiation, these cells develop functional tight junctions and express key intestinal enzymes and transporters, thereby providing a suitable platform for comprehensive evaluation of polyphenol absorption kinetics and phase I and II metabolism (including deglycosylation and conjugation reactions) [86,87]. ICH M9 guidance adopted by European Medicines Agency (EMA) and the United States Food and Drug Administration (FDA) recognizes validated and standardized Caco-2 assays for assessing intestinal drug permeability in the context of Biopharmaceutical Classification System (BCS) [88,89,90]. This cell line is also used to assess epithelial barrier protection, the regulation of tight junctions, and the modulation of NF-κB in the mitigation of intestinal inflammation [91].

Jurkat T cells are crucial for evaluating adaptive immune responses, and THP-1 monocytic cells serve as a model for innate immunity; when stimulated with LPS, they produce tumor necrosis factor-alpha (TNF-α), IL-6, IL-1β, cyclooxygenase-2 (COX-2), nitric oxide (NO), and prostaglandin E_2_ (PGE_2_), while also generating intracellular ROS [92].

Scientific literature indicates that walnut polyphenols activate antioxidant defense mechanisms, particularly the Nrf2/ARE pathway. They may also attenuate inflammatory responses by inhibiting NF-κB phosphorylation, modulating MAPK signaling cascades, and suppressing the CD14/TLR4/MyD88 signaling axis [29,30,93,94].

Thus, using Caco-2, Jurkat, and THP-1 cell lines provides a comprehensive in vitro framework for assessing the antioxidant and anti-inflammatory bioactivities of WSLE and WPLE in relation to the gut-immunity axis.

#### 2.5.1. Cell Viability Assays

**Figure 6:**
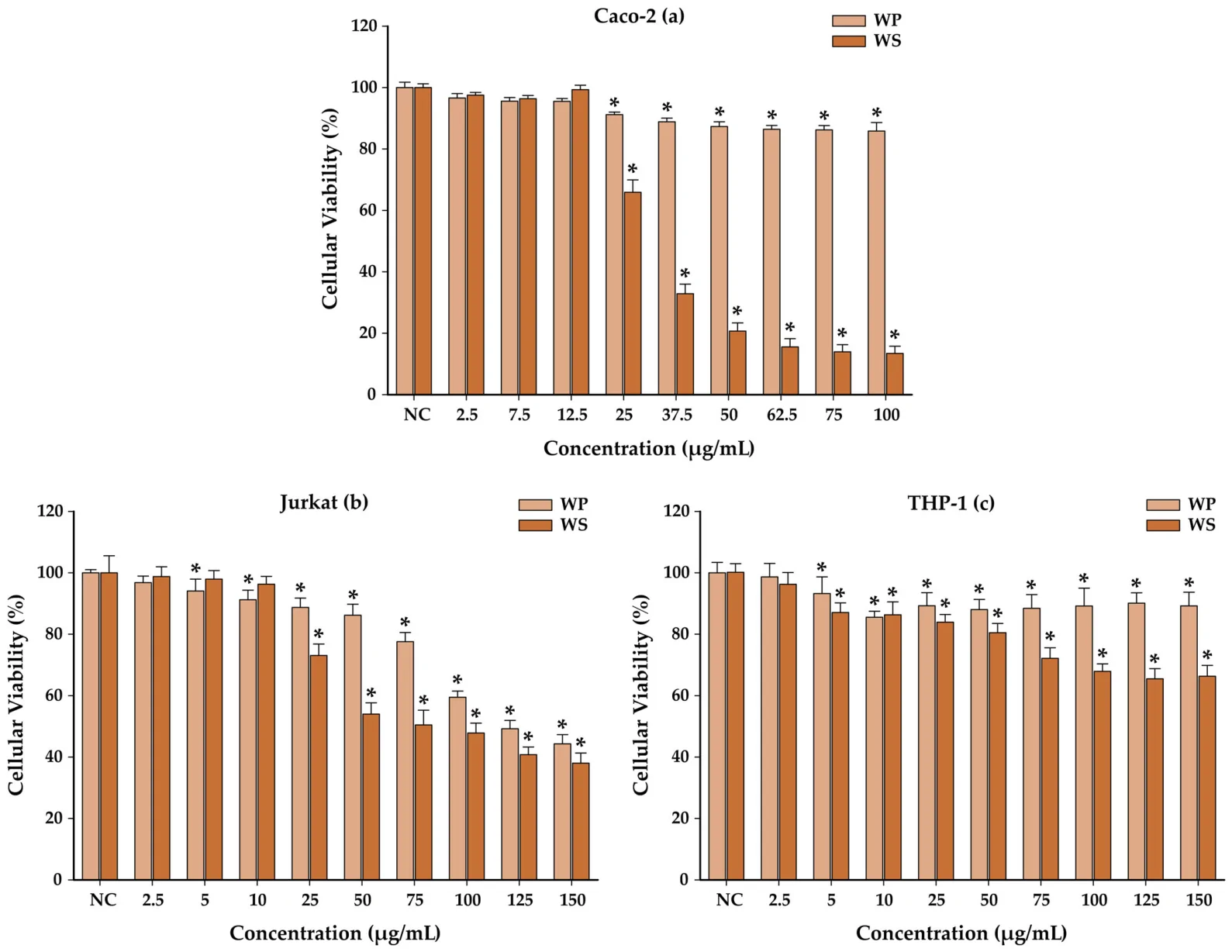
Cell viability of WPLE and WSLE was evaluated by the Alamar Blue assay in the Caco-2 intestinal cells (**a**), in Jurkat cells (**b**), and in THP-1 (**c**) immune cells after a 24 h exposure. Results are expressed as means ± SD (*n* = 3) relative to the negative control (NC, 100%). Asterisks (*) indicate statistically significant differences from the negative control (ANOVA + post hoc Holm–Šídák test at *p* < 0.05).

Figure 6 shows the results of cell viability assays performed with WSLE and WPLE on the Caco-2, Jurkat, and THP-1 cell lines. The Alamar Blue assay was used to determine cell viability by measuring resazurin reduction as an indicator of metabolic activity.

In the cell-free controls, neither WSLE nor WPLE produced a measurable change in the resazurin assay signal under the experimental conditions, indicating no detectable direct interference with the assay readout.

To facilitate the comparative assessment of biological potency, the half-maximal inhibitory concentration (IC_50_) values were determined for both extracts across the evaluated cell lines (Table 6).

Cell viability is a key parameter for designing subsequent bioactivity assays and for assessing the cytocompatibility of the extracts under the tested in vitro conditions, with a view to their potential use as functional agents or nutraceuticals. In this study, Alamar Blue cell viability assays on Caco-2, Jurkat, and THP-1 cells (Figure 6a–c, Table 6) revealed cell-line-specific changes in viability across the tested concentration range.

In Caco-2 intestinal cells, WPLE was consistently better tolerated than WSLE, indicating a broader cytocompatible concentration range (2.5–100 µg/mL). By contrast, WSLE reduced cell viability more at higher concentrations, limiting its application to lower doses (2.5–12.5 µg/mL) under the tested conditions (Figure 6a, Table 6).

In Jurkat cells, both extracts induced a concentration-related decrease in metabolic activity across the tested concentration range (Figure 6b, Table 6). WSLE produced a marked reduction at higher concentrations (100–150 µg/mL) and a moderate reduction at intermediate doses (25–75 µg/mL). WPLE also showed a dose-dependent effect, though less pronounced, with cell viability remaining above 55% even at 100 µg/mL. Both WSLE and WPLE were well tolerated by THP-1 monocytic cells, with IC_50_ values exceeding 150 µg/mL for both extracts (Figure 6c, Table 6).

A comparative analysis of our results with data reported in the literature [29,95,96,97] reveals that the effects of the WS and WP extracts on cell viability depend on the cell line tested, the malignant or non-malignant phenotype of the cells, and the method used to prepare the extracts (their phytochemical composition). For WS extracts tested on both malignant (A549, A172, and T47D KBluc) and non-malignant (HGF, HFF-1, rBM-MSCs, and C2C12-IR) cell lines, the effects on cell viability do not appear to be limited to malignant cells. Certain non-malignant intestinal cells showed sensitivity to WSE comparable to, or even greater than, that observed in fibroblast or skeletal muscle cells. Compared to other plant-derived matrices, including berry extracts, our WSLE exhibited a relatively strong reduction in metabolic activity in intestinal cells [47].

In the present study, WPLE showed a more favorable cytocompatibility profile than WSLE, particularly in Caco-2 and THP-1 cells (IC_50_ > 100 µg/mL) after 24 h of exposure (Table 6). A WP extract prepared in ethanol by maceration exhibited similar effects on Caco-2 cell viability (IC_50_ of 250 µg/mL) after prolonged exposure (48–72 h) [38].

#### 2.5.2. Cellular Antioxidant Activity of Extracts

**Figure 7:**
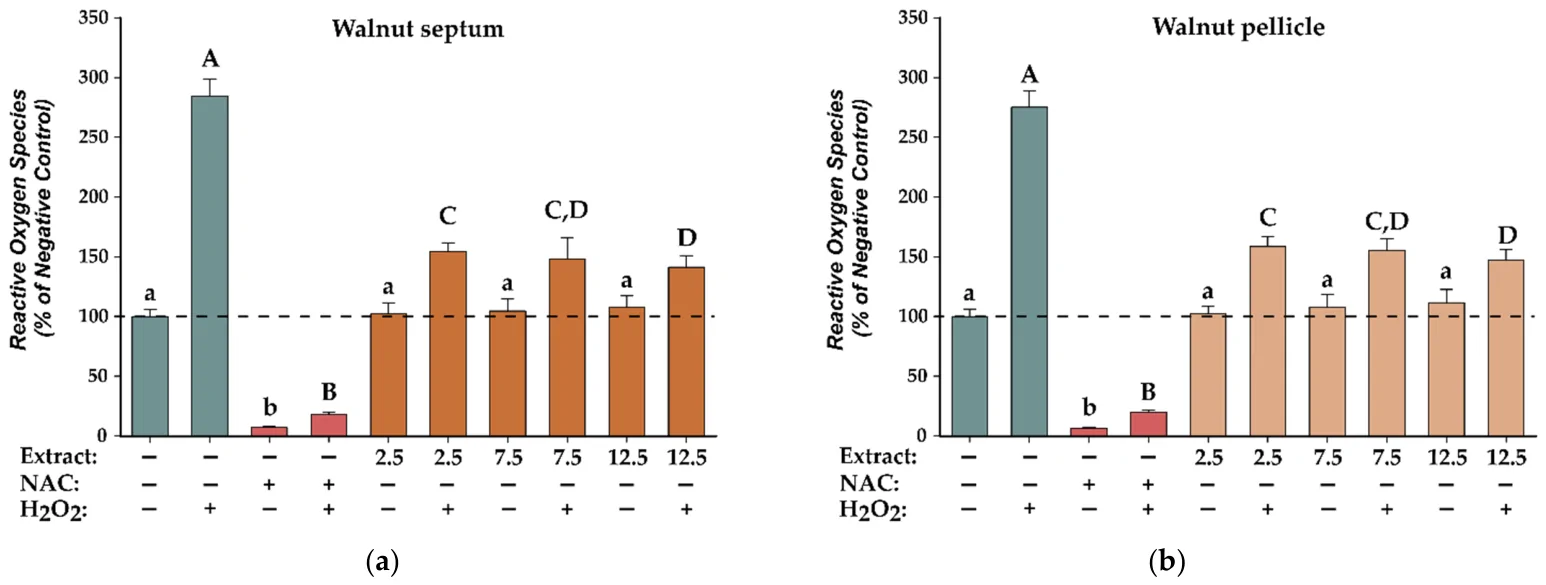
Effects of WSLE (**a**) and WPLE (**b**) on DCF fluorescence in Caco-2 intestinal cells, assessed using the DCFH-DA assay. Cells were incubated with the extracts (2.5, 7.5, and 12.5 µg/mL) or NAC (5 mM) for 24 h and subsequently loaded with 50 µM DCFH-DA. The DCF fluorescence signal was assessed under non-stimulated conditions and after stimulation with 250 µM H_2_O_2_ for 2 h. Data are presented as relative mean values ± SD (*n* = 3). Values are expressed relative to the negative control (100%), indicated by the dashed line. Grey-blue bars represent the negative control and the H_2_O_2_-stimulated control, red bars represent NAC-treated cells, and orange bars represent extract-treated cells (dark orange, WSLE; light orange, WPLE). Different lowercase letters (a,b) under non-stimulated conditions and uppercase letters (A–D) under H_2_O_2_-stimulated conditions indicate statistically significant differences, as determined by one-way ANOVA followed by the Holm–Šídák post hoc test (*p* < 0.05).

Figure 7 illustrates the antioxidant effects of WSLE and WPLE on Caco-2 cells.

The DCFH-DA assay showed that both WSLE and WPLE attenuated the H_2_O_2_-induced increase in intracellular DCF fluorescence in Caco-2 cells across the tested concentration range (2.5 to 12.5 µg/mL), with no evidence of pro-oxidant behavior under the experimental conditions. Under basal conditions, both WSLE and WPLE treatments maintained DCF fluorescence signals close to those of the untreated control, ranging from 99% to 109% of the baseline value (Figure 7a,b).

After the H_2_O_2_ oxidative challenge, DCF fluorescence in Caco-2 cells increased significantly, reaching approximately 2.8 times the unstimulated control value (Figure 7a,b). The positive control, N-acetylcysteine (NAC), markedly reduced the H_2_O_2_-induced DCF fluorescence signal to below the unstimulated control level (Figure 7a,b).

Pre-treatment with WSLE or WPLE significantly attenuated the H_2_O_2_-induced increase in DCF fluorescence. Both extracts reduced the H_2_O_2_-induced fluorescence signal by approximately 50% to a comparable extent at all tested concentrations (Figure 7a,b). Specifically, relative to the H_2_O_2_-stimulated cells, WSLE lowered the 2′,7′-dichlorofluorescein (DCF) signal by 46%, 48%, and 50%, while WPLE reduced it by 42%, 44%, and 46% at concentrations of 2.5, 7.5, and 12.5 µg/mL, respectively. At the highest concentration tested (12.5 µg/mL), the DCF signal corresponded to 141% and 147% of the unstimulated baseline for WSLE and WPLE, respectively. A direct comparison of the two extracts (Figure 7a,b) indicated that WSLE and WPLE produced a comparable attenuation of the DCF fluorescence signal in Caco-2 cells, with no evidence of an increase in the signal beyond that induced by H_2_O_2_.

The antioxidant effects of the two extracts may be associated with the presence of phenolic compounds and their potential to participate in several chemical and cellular processes. Structurally, EA is a chromene-dione polyphenol characterized by four hydroxyl and two lactone groups. These functional groups can facilitate free-radical scavenging through hydrogen-atom and electron-transfer mechanisms, while the deprotonated phenolic groups allow for the chelation of redox-active transition metals [98]. Thus, ellagic acid has been described as capable of acting as both a primary antioxidant, through the donation of phenolic hydrogen atoms, and a secondary antioxidant, through the inhibition of hydroxyl-radical formation and chelation of redox-active metals such as iron and copper [25,28]. Previous studies have also suggested that EA may influence ROS- and RNS-generating enzymes and redox-sensitive signaling pathways, including the Nrf2/ARE pathway, with subsequent effects on antioxidant and cytoprotective enzymes such as glutathione peroxidase (GPx), catalase (CAT), superoxide dismutase (SOD) [25,28], and heme oxygenase-1 (HO-1) [99]. In living organisms, urolithins—EA metabolites generated by the gut microbiota—may also contribute to the biological effects associated with EA. Because some urolithins exhibit higher tissue bioavailability compared to the parent compound, they have been proposed as important mediators of EA’s systemic bioactivity [98,100].

The galloyl moiety of GA, characterized by a vicinal trihydroxyl (pyrogallol) configuration, may account for its low redox potential and its metal-chelating and radical-scavenging capacity. Furthermore, this structural feature has been associated with attenuation of the NF-κB-mediated pro-inflammatory signaling cascade in previous studies [75]. Similarly, other phenolic acids, flavan-3-ols, and quercetin glycosides have been reported to exhibit cellular antioxidant-related effects through mechanisms that may include direct redox reactions, metal chelation, and modulation of redox-sensitive signaling pathways, particularly Nrf2/ARE [25]. Thus, the antioxidant potential of catechin is closely linked to the ortho-dihydroxy (catechol) configuration of the B-ring and the presence of the 3-hydroxyl group on the C-ring. This molecular structure can facilitate radical-scavenging reactions, transition-metal chelation, and the formation of resonance-stabilized phenoxyl radicals [101]. Beyond its direct antioxidant capacity, catechin has been reported to influence redox-sensitive cellular signaling, including the Nrf2/ARE pathway, and to attenuate NF-κB-mediated pro-inflammatory signaling [102].

Procyanidins B1 and B3—dimers of (epi)catechin units linked via B-type (C4 → C8) interflavan bonds—contain an increased density of reactive hydroxyl groups, which may contribute to their free radical scavenging and protein/metal-chelation capacities compared to their monomeric counterparts [101]. Despite their limited systemic bioavailability, these dimers may exert direct biological effects within the intestinal epithelium and may subsequently be catabolized by the gut microbiota into more bioavailable phenyl-γ-valerolactone derivatives [102].

The similar magnitude of DCF fluorescence attenuation in Caco-2 cells for the two extracts, WSLE and WPLE (Figure 7), contrasts with the results of the DPPH, ABTS, and FRAP assays (Table 2). In these cell-free assays, WPE consistently exhibited higher antioxidant capacity than WSE, with approximately double the radical-scavenging activity in the DPPH and ABTS assays and substantially higher FRAP values. This divergence between chemical and cellular assays indicates that the cellular DCF fluorescence response cannot be inferred solely from chemical radical-scavenging capacity measured in cell-free systems. Factors such as solubility differences in the culture medium, cellular uptake, intracellular distribution, phase II metabolism, and interactions with membrane components or proteins may have contributed to the distinct responses observed in the cell-free and cellular assays. The comparable attenuation of DCF fluorescence produced by WSLE and WPLE, despite WSLE’s lower performance in the cell-free assays, may reflect differences in matrix composition, cellular availability, and/or interactions among phenolic subclasses.

Our results are consistent with previous reports describing cellular antioxidant effects of walnut septum extracts. Using the same extraction solvent (water:acetone 50:50, *v*/*v*) and DCFH-DA assay, Rusu et al. (2020) [29] demonstrated that WSLE statistically significantly reduced both basal and H_2_O_2_-induced DCF fluorescence in A549, MCF-7, and HGF cells, supporting a remarkable antioxidant effect across different cell types. Similarly, Hu et al. (2021) [103] reported that hot water and 70% ethanol walnut septum extracts attenuated oxidative-stress-related responses in HepG2 cells, consistent with their high DPPH- and ABTS-scavenging activities.

Given the central role of cellular redox balance in preserving intestinal barrier integrity and shaping downstream immune responses, the observed attenuation of H_2_O_2_-induced DCF fluorescence may be relevant. Several studies have shown that dietary antioxidants may exert anti-inflammatory effects and influence the release of cytokines and other pro-inflammatory mediators. Complex mechanisms may be involved, including the reciprocal interaction between the Nrf2/ARE and NF-κB signaling pathways, which participate in the regulation of oxidative-stress responses and inflammation [100]. These findings motivated further research through preliminary studies on the ability of WSLE and WPLE to modulate inflammatory processes.

#### 2.5.3. Exploratory Assessment of Immunomodulatory Effects

This exploratory experiment aimed to assess whether WSLE and WPLE could modulate LPS-induced cytokine release across three cell lines: Caco-2, Jurkat, and THP-1. LPS stimulation of Caco-2 and Jurkat cells did not produce any measurable increase in cytokine production. Although earlier studies suggested these cells might respond to LPS, a more detailed literature review indicated that this response is often limited or absent because of low or functionally incomplete expression of TLR4 and its required co-receptors, MD-2 and CD14 [104,105].

Among the three tested cell lines, only THP-1 exhibited a statistically significant increase in IL-8 levels following LPS stimulation (*p* < 0.05 vs. control). The LPS concentration used in this study (100 ng/mL) was associated with the best cell viability in THP-1 cells and an adequate inflammatory response and was therefore considered the optimal test concentration [106]. Widdrington et al. (2018) found that exposing THP-1 cells to 100 ng/mL LPS for 72 h did not adversely affect their viability [107]. Preliminary assays of THP-1 cell viability at LPS concentrations up to 100 ng/mL in this study showed that LPS did not reduce cell viability within the concentration range investigated (Figure S1). In contrast, a modest increase in the metabolic/viability signal was observed in the presence of LPS, including at the highest concentration tested (100 ng/mL). Similar results are reported in the literature by Ubanako et al. (2019) [108]. Consequently, the anti-inflammatory screening was limited to a single responsive readout: LPS-induced IL-8 release in THP-1 cells. Therefore, the findings should be interpreted strictly within this limited, exploratory framework.

**Figure 8:**
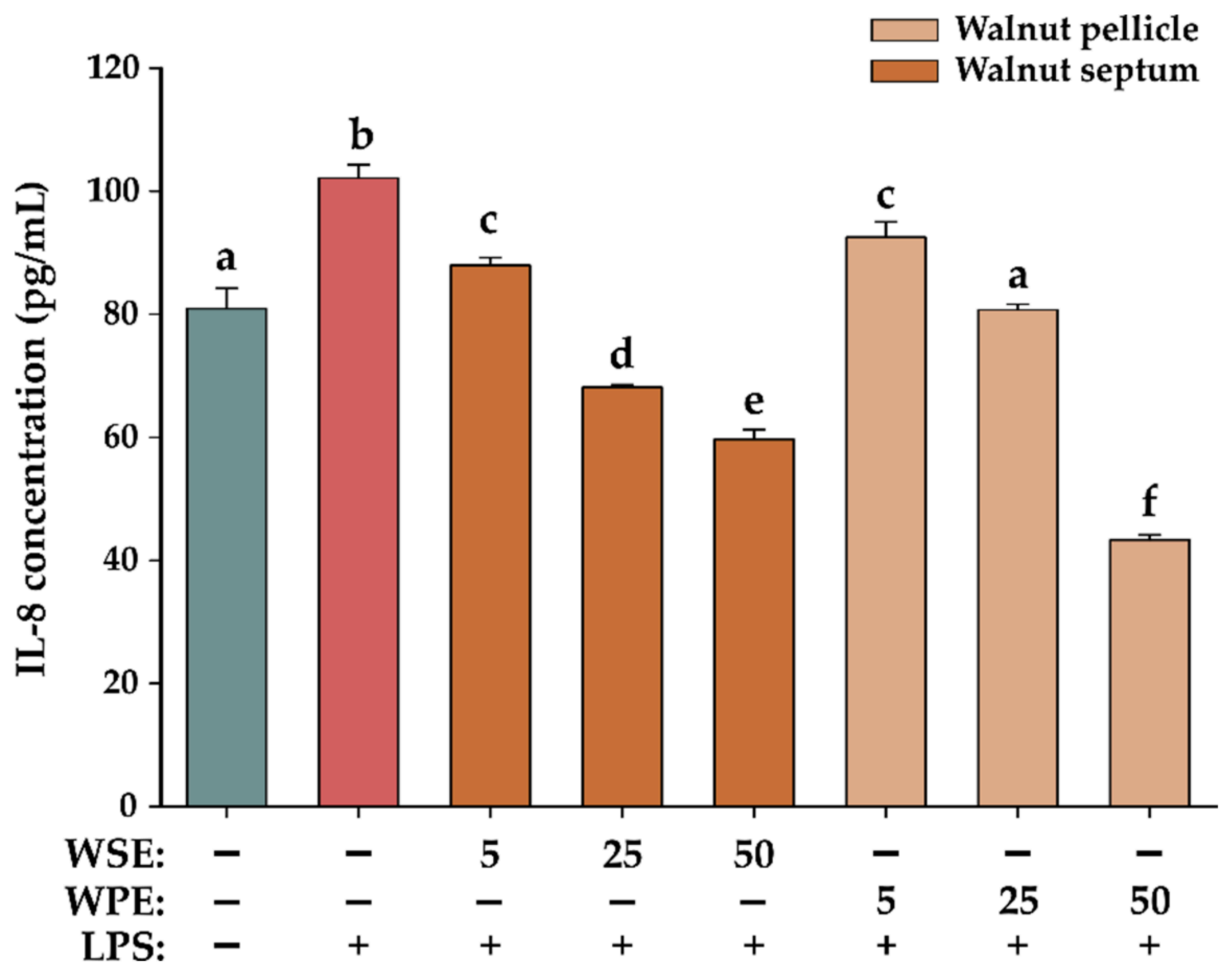
Effect of WSLE and WPLE on LPS-induced IL-8 secretion in THP-1 cells. THP-1 cells were exposed for 24 h to WSLE or WPLE at concentrations of 5, 25, or 50 µg/mL in the presence of 100 ng/mL LPS. IL-8 concentrations were subsequently determined in the cell-culture supernatants. The grey-blue bar represents untreated control cells (without LPS), the red bar represents cells stimulated with LPS alone, and the dark orange and light orange bars represent LPS-stimulated cells treated with WSLE and WPLE, respectively. Data are expressed as the mean ± SD (*n* = 3). Different letters indicate statistically significant differences in IL-8 concentrations (repeated-measures ANOVA with the independent experiment as the blocking factor, followed by the Holm–Šídák post hoc test; *p* < 0.05).

Both WSLE and WPLE reduced LPS-induced IL-8 secretion in THP-1 cells (Figure 8). All tested concentrations (5, 25, and 50 µg/mL) resulted in significantly lower IL-8 levels than the LPS-stimulated control (*p* < 0.05), and at 25 and 50 µg/mL, IL-8 fell to, or even below, the basal levels of the unstimulated control.

The results indicated that both WSLE and WPLE reduced LPS-induced IL-8 release in THP-1 cells (Figure 8). These observations are consistent with previous reports on walnut septum preparations. Rusu et al. (2020) [29] reported that WSLE reduced IL-6, IL-8, and IL-1β in LPS-stimulated normal human gingival fibroblasts at non-cytotoxic doses. Similarly, Wang et al. (2018) [109] showed that isolated WSLE constituents, particularly gallic acid and ethyl gallate, markedly suppressed inflammatory responses in LPS-stimulated RAW 264.7 macrophages. Although these studies provide supportive context, the present data are limited to one cell line model and the measurement of a single pro-inflammatory biomarker; therefore, they do not, by themselves, demonstrate a broad anti-inflammatory effect. Furthermore, IL-8 levels were not normalized to the viable cell number, total cellular protein, or metabolic activity measured in each well after treatment. Although equal initial cell densities and extract concentrations previously identified as non-cytotoxic were used, treatment-related differences in cell number or metabolic state cannot be entirely excluded. Consequently, the absence of post-treatment normalization represents a residual limitation, and the observed reductions in IL-8 release should be interpreted as preliminary evidence of an immunomodulatory effect. Differences in potency and cytokine profiles across models likely reflect cell type-specific responses, extract composition, exposure conditions, and the nature of the inflammatory stimulus.

Taken together, the distinct phytochemical profiles of WSLE and WPLE position both extracts as promising sources of natural antioxidant compounds, with preliminary evidence of an effect on LPS-induced IL-8 release in THP-1 cells. Given the absence of post-treatment normalization of IL-8 levels to viable cell number, total cellular protein, or metabolic activity, this observation requires further confirmation using normalized cytokine measurements and additional inflammatory biomarkers. WSLE, characterized by catechins, procyanidins, and quercetin glycosides, showed antioxidant-related effects comparable to WPLE in the cellular oxidative-stress model. In contrast, the notable levels of ellagic acid, gallic acid, and castalagin in WPLE may contribute to its stronger antioxidant capacity in cell-free assays. These findings support the potential valorization of walnut septum and pellicle as biologically active by-products, while also highlighting the need for further mechanistic and functional validation.

The study provides new evidence for the bioactivity of certain extracts prepared from walnut by-products. To the best of our knowledge, this is the first comprehensive phytochemical analysis of WS and WP from a region in Romania, performed using a sensitive LC-QTOF-MS/MS method. It is also among the first studies to directly compare the antioxidant-related and preliminary immunomodulatory effects of walnut septum and pellicle extracts in human cell-based models.

The results are exploratory and open new perspectives for future research. Further studies using differentiated intestinal barrier models, epithelial–immune cell co-cultures, and isolated compounds are required to clarify the mechanisms involved and to validate the biological relevance of these extracts. In addition, experiments examining the metabolically transformed polyphenol derivatives, such as urolithins derived from ellagitannins, would be valuable, given their potential contribution to the biological effects associated with ellagic acid-rich matrices. These efforts will help clarify the roles of WSLE and WPLE in maintaining gut-associated redox balance and immune homeostasis.

### 2.6. Study Limitations

Several limitations should be considered when interpreting the findings of the current study.

First, the WSLE and WPLE were obtained from a single orchard, harvest year, and pooled plant-material batch. Therefore, the study does not account for variability due to cultivar, genotype, geographic area, climatic conditions, post-harvest handling, or storage conditions. The phytochemical composition and biological activity reported in this study should therefore be considered as representative of the investigated batch rather than as general characteristics of WSLE and WPLE. In addition, spectrophotometric assays were performed using crude hydro-acetonic extracts, whereas LC–MS profiling and cell-based assays used lyophilized preparations. Since lyophilization may affect the stability or relative abundance of certain phenolic constituents, and no direct compositional comparison between the crude and lyophilized preparations was performed, the relationship between the LC–MS profiles of the lyophilized extracts and the antioxidant activities of the crude extracts should be regarded as indicative rather than strictly mechanistic.

Second, Caco-2 cells were used under short-term, non-differentiated culture conditions and were not grown as polarized monolayers on permeable supports. Therefore, although Caco-2 cells were used as an intestinal epithelial model, the experiments presented in this study were designed mainly to assess cytocompatibility and cellular redox responses rather than intestinal barrier function.

Third, the evaluation of immunomodulatory activity was exploratory, as it does not provide definitive conclusions but rather lays the groundwork for further detailed research. LPS stimulation did not induce a sufficiently robust pro-inflammatory response in Caco-2 or Jurkat cells. In contrast, it induced a modest but statistically significant increase in IL-8 secretion in THP-1 cells under the experimental conditions used. Accordingly, the observed reduction in LPS-induced IL-8 secretion should be regarded as preliminary evidence of anti-inflammatory potential rather than as a comprehensive characterization of immunomodulatory activity. Additional studies using cell-type-appropriate stimulation protocols and a broader panel of inflammatory mediators and signaling endpoints would be necessary to define the underlying mechanisms more precisely. Moreover, IL-8 concentrations were not normalized to viable cell number, total cellular protein, or metabolic activity after treatment. Although cell viability in the presence of LPS was verified and all wells were seeded at identical densities, equal seeding does not guarantee equal viable cell numbers or metabolic states after 24 h. Therefore, the observed reduction in IL-8 release should be interpreted with caution.

Fourth, intracellular oxidative stress was assessed using the DCFH-DA assay, which provides a useful overall indicator of changes in cellular redox status but does not distinguish between direct effects of radical scavenging and modulation of endogenous antioxidant pathways. Therefore, the observed reduction in fluorescence should be considered evidence of attenuated oxidative stress rather than evidence of a specific antioxidant mechanism. Furthermore, the Alamar Blue assay measures resazurin reduction as an indicator of cellular metabolic activity rather than cell number or cell death. Although cell-free controls showed no direct interaction between the extracts and resazurin under the experimental conditions, this assay alone cannot fully distinguish changes in metabolic activity from changes in viable cell number. Alternative cell viability assays, such as those measuring DNA content through fluorescent dye binding, are needed to better elucidate the underlying mechanisms.

Finally, the molecular docking and molecular dynamics simulations provide hypothesis-generating evidence that selected major phenolic constituents may interact with KEAP1. However, these computational findings do not demonstrate intracellular KEAP1 binding or activation of the Nrf2/ARE pathway. Experimental confirmation using pathway-specific approaches, such as assessment of Nrf2 nuclear translocation and expression of downstream targets (for instance, HO-1 or NAD(P)H quinone oxidoreductase 1 (NQO1)), would be necessary to establish a causal mechanistic relationship. Likewise, because the biological effects were evaluated using complex extracts rather than isolated bioactive compounds, the contribution of individual constituents and potential synergistic or antagonistic interactions cannot be conclusively established. Matrix composition, compound solubility, cellular uptake, and concentration-dependent responses may additionally influence the measured activity. Therefore, the results should be interpreted as reflecting the overall activity of the extracts rather than the effect of individual compounds. Further studies involving fractionation, isolated-compound testing, and more physiologically relevant co-culture or in vivo models are therefore necessary.

## 3. Materials and Methods

### 3.1. Plant Matrices

Walnuts (*Juglans regia* L.) were harvested in September 2024 from an orchard located in Cluj-Napoca, Romania (46°46′30″ N, 23°35′30″ E), and stored in a dark, ventilated environment at approximately 0 °C until processing. To ensure traceability and standardization of the plant material, the walnuts were collected directly from the orchard and identified at the Department of Pharmaceutical Botany by Ana-Maria Vlase, who assigned them the specimen number 35.1.1.1/2024. The plant material was then processed manually in the laboratory to minimize potential cross-contamination and uncontrolled degradation associated with complex industrial processing. The septum and pellicle were manually separated from the shells and walnut kernels, respectively, and dried at room temperature. Both the septum and pellicle were ground for 5 min using a coffee grinder (Argis, RC-21, Electroarges SA, Curtea de Argeș, Romania). The resulting powders, hereafter referred to as WS and WP, were passed through a 200 µm Retsch sieve to obtain a uniform particle-size fraction. A total of approximately 2 kg (2002 g) of in-shell walnuts were processed, corresponding to approximately 172 individual walnuts with masses ranging from 6.88 to 17.90 g (mean ± SD: 11.64 ± 2.15 g). This material yielded 835.44 g of kernels, representing 41.73% of the total in-shell walnut mass. After manually separating the kernel components, 95.32 g of WS were isolated, accounting for 4.76% of the in-shell walnut mass and 11.41% of the kernel mass. In addition, 42.44 g of WP were recovered, corresponding to 2.12% of the in-shell walnut mass and 5.08% of the kernel mass.

The moisture content in WS and WP was determined gravimetrically using a laboratory oven (Conterm Oven, JP Selecta S.A., Barcelona, Spain), following the method described by Toǧrul and Arslan (2007) [110]. Samples were dried at 105 ± 2 °C until constant mass (± 0.01 g) was achieved and were weighed in triplicate using an electronic analytical balance (Discovery DV215CD, Ohaus Corp., Parsippany, NJ, USA). The moisture contents were 8.625% for WS and 5.484% for WP. All quantitative results are expressed on a dw basis to standardize compound concentrations independently of residual moisture content. The dried WS and WP powders were subsequently subjected to extraction and lyophilization, as described in Section 3.3.

### 3.2. Reagents

All the reagents and standards used in this study were of analytical grade. The reagents and solvents—including 2,2-diphenyl-1-(2,4,6-trinitrophenyl)hydrazine (DPPH), 2,4,6-tris(2-pyridyl)-s-triazine (TPTZ), 6-hydroxy-2,5,7,8-tetramethylchromane-2-carboxylic acid (Trolox, 97%), acetone, N-acetyl cysteine (NAC), aluminum chloride, ammonium persulfate, diammonium 2,2′-azino-bis(3-ethylbenzothiazoline-6-sulfonate) (ABTS), 2′,7′-dichlorodihydrofluorescein diacetate (DCFH-DA), DMSO, ethanol, ferric chloride, fetal bovine serum (FBS), penicillin, streptomycin, glacial acetic acid, L-glutamine, GlutaMAX, insulin (cell culture tested), methanol, non-essential amino acids (NEAA), resazurin, sodium acetate trihydrate, sodium carbonate, trypsin, and vanillin (99%)—were purchased from Sigma-Aldrich (Schnelldorf, Germany). The Folin–Ciocâlteu reagent and hydrochloric acid (37%) were purchased from Merck (Darmstadt, Germany).

For quantitative analysis, 75 phenolic compound reference standards (Table S2) were purchased from Sigma Aldrich Chemie GmbH (Schnelldorf, Germany), Carl Roth GmbH (Karlsruhe, Germany), Merck (Darmstadt, Germany), Supelco (Bellefonte, PA, USA), and Extrasynthese (Genay, France). MS-grade methanol and MS-grade acetic acid were obtained from Merck KGaA (Darmstadt, Germany). Ultrapure water was obtained using a Milli-Q Direct 8 water purification system (Millipore, Bedford, MA, USA).

Dulbecco’s Modified Eagle’s medium (DMEM) for Caco-2 cultures was purchased from Thermo Fisher Scientific (Waltham, MA, USA). 4-(2-hydroxyethyl)-1-piperazine-ethanesulfonic acid (HEPES) and phosphate-buffered saline (PBS) were supplied by Sigma Chemical Co. (St. Louis, MO, USA). The Roswell Park Memorial Institute (RPMI) medium for Jurkat cells was obtained from Biowest (Nuaillé, France).

### 3.3. Extract Preparation

The extraction process was conducted under conditions previously optimized by our group to maximize polyphenolic recovery and antioxidant activity of the resulting extracts [28]. The WS and WP powders were separately mixed with a water/acetone solution (50:50, *v*/*v*) at a solid-to-solvent ratio of 1:10 (*w*/*v*). The UTE was carried out by turbo-extraction using an Ultra-Turrax homogenizer (T18; IKA Labortechnik, Staufen, Germany) for 2 min, followed by vortex agitation (RX-3; Velp Scientifica, Usmate Velate, Italy) for an additional 2 min. The mixtures were centrifuged at 3000 rpm for 15 min, and the supernatants were carefully collected. Aliquots of 2 mL of crude WS and WP extracts (referred to as WSE and WPE, respectively) were stored and analyzed to determine the TPC, TFC, CTC, and the antioxidant activity using the Trolox equivalent antioxidant capacity (TEAC), DPPH, and ferric reducing antioxidant power (FRAP) assays.

Acetone was removed by rotary evaporation (Hei-VAP, Heidolph Instruments GmbH & Co., Schwabach, Germany). The remaining aqueous extracts were lyophilized using the Advantage 2.0 system (SP Scientific, Warminster, PA, USA). Until LC-HRMS analysis or in vitro biological evaluation, the resulting lyophilized extracts, which were designated as WSLE and WPLE, were stored in the dark at room temperature.

Prior to chromatographic analysis, the appropriate quantities of WSLE and WPLE were dissolved in a 50:50 (*v*/*v*) water/acetone solution. In the LC-HRMS system was injected 1 µL of the reconstituted extracts. All assays were conducted in triplicate. For the cell-based assays, the appropriate quantities of WSLE and WPLE were solubilized in DMSO and diluted with cell culture medium prior to the analyses, unless otherwise specified.

The crude hydro-acetonic extracts (WSE and WPE) were used for general characterization by spectrophotometric determinations (TPC, TFC, and CTC) and abiotic antioxidant assays (ABTS, DPPH, and FRAP), whereas the solvent-free lyophilized extracts (WSLE and WPLE) were employed for LC-HRMS phytochemical profiling and all cell-based assays. Lyophilized, acetone-free extracts were required for the biological experiments because residual extraction solvent in the crude extracts may be cytotoxic to cells. In addition, lyophilization provides standardized, storable material, improves the stability of bioactive compounds, and prevents solvent interference during biological analyses.

### 3.4. Quantitative Determinations of Total Bioactive Compounds

#### 3.4.1. Total Phenolic Content

The TPC was determined using the Folin–Ciocâlteu spectrophotometric assay [111,112]. The crude extracts of WS and WP were first dissolved in methanol to prepare stock solutions, followed by dilution with distilled water to achieve the desired working concentrations. For each measurement, 20 µL of diluted extract was mixed with 100 µL of Folin–Ciocâlteu reagent (diluted 1:10 *v*/*v*). After a 3 min incubation period, 80 µL of 7.5% (*w*/*v*) sodium carbonate solution was added to achieve the final reaction volume in 96-well microplates. The mixtures were incubated in the dark at room temperature for 30 min, after which absorbance was measured at 760 nm using a Synergy HT Multi-Detection Microplate Reader (BioTek Instruments, Inc., Winooski, VT, USA) against a solvent blank. Gallic acid served as the reference standard, and a calibration curve was prepared over a concentration range of 0.01–0.16 mg/mL (R^2^ = 0.9997). TPC values were calculated from the calibration curve and expressed as gallic acid equivalents per gram of dry plant material (mg GAE/g dw).

#### 3.4.2. Total Flavonoid Content

The TFC was determined using an aluminum chloride colorimetric assay, as previously described [29,113]. Briefly, 100 µL of each appropriately diluted extract was combined with 100 µL of a 2% (*w*/*v*) aqueous AlCl_3_ solution in 96-well plates and incubated in the dark at room temperature for 15 min. Absorbance was then measured at 420 nm against a solvent blank. A calibration curve was constructed using quercetin as the reference standard over a concentration range of 0.01–0.16 mg/mL (R^2^ = 0.9986). TFC values were expressed as milligrams of quercetin equivalents per gram of dried plant material (mg QE/g dw).

#### 3.4.3. Condensed Tannin Content

The CTC was assessed using a modified version of the vanillin assay, as previously reported [28]. Briefly, 250 µL of a 0.5% vanillin solution (prepared in 4% concentrated HCl in methanol) was added to 50 µL of extract in 96-well plates. The mixtures were then incubated in the dark at 30 °C for 20 min. Absorbance was subsequently measured at 500 nm against a solvent blank. A calibration curve was constructed using catechin as the reference standard, prepared over a concentration range of 0.1–1.6 mg/mL (R^2^ = 0.9997). CTC values were expressed as catechin equivalents per gram of dried plant material (mg CE/g dw).

### 3.5. Antioxidant Activity Assays

The antioxidant activities of crude extracts were assessed using the ABTS radical cation scavenging assay, the DPPH radical-scavenging assay, and the FRAP assay. The results were calculated and expressed as Trolox equivalents per gram of dried plant material (mg TE/g dw).

#### 3.5.1. ABTS Radical Cation Scavenging Activity

The ABTS radical cation scavenging assay was employed to determine the total antioxidant activity (TAA) of the WSE and WPE, following the TEAC method as previously described [28,29,113]. Briefly, 10 µL of Trolox standards or appropriately diluted samples were mixed with 190 µL of ABTS•^+^ working solution in 96-well plates. Each mixture was incubated for 30 min in the dark with gentle shaking, and the absorbance was recorded at 734 nm. The scavenging activity against the ABTS radical cation was assessed and used to construct a Trolox calibration curve generated within the concentration range of 0.05–0.1 mg/mL (R^2^ = 0.9996).

#### 3.5.2. DPPH Radical-Scavenging Activity

The WSE and WPE antiradical activity was assessed using a method previously described [28]. The DPPH free radical scavenging capacity was determined in a 96-well plate by mixing 30 µL of the sample solution with 200 µL of a 0.008% (*w*/*v*) methanolic DPPH solution, followed by incubation for 30 min in the dark. The absorbance at 517 nm was measured against a solvent blank. The DPPH scavenging capacity was calculated in comparison with Trolox as the reference standard. A calibration curve was generated within the concentration range of 0.025–0.5 mg/mL (R^2^ = 0.9988).

#### 3.5.3. FRAP Assay

The reducing abilities of WSE and WPE were evaluated by quantifying the formation of the blue Fe^2+^–TPTZ complex, which arises from the reduction of Fe^3+^–TPTZ. This technique was modified from a previously outlined procedure [114]. In brief, 25 µL of the sample was mixed with 175 µL of a freshly prepared FRAP reagent. This reagent consisted of 300 mM acetate buffer (pH 3.6), 10 mM TPTZ in 40 mM HCl, and 20 mM FeCl_3_·6H_2_O in 40 mM HCl, combined in a 10:1:1 (*v*/*v*/*v*) ratio and placed in a 96-well microplate. The mixture was kept in the dark at ambient temperature for 30 min, then absorbance was measured at 593 nm. Trolox served as an external standard, and a calibration curve was created using a concentration range of 0.031–0.1 mg/mL (R^2^ = 0.9991).

### 3.6. Phytochemical Analysis of Bioactive Compounds by High-Resolution LC-MS (LC-HRMS)

Chromatographic separation was performed using a Shimadzu Nexera X3 HPLC system (Shimadzu Corporation, Kyoto, Japan), which included two LC-40D X3 pumps with a degasser, a SIL-40C X3 autosampler, and a CTO-40S column oven. The LC system was coupled with a Shimadzu QTOF 9030 mass spectrometer. Sample preparation used a Sigma 204 centrifuge (Osterode am Harz, Germany), an Analytical Plus Balance (Mettler-Toledo, Greifensee, Switzerland), and an Elma Transsonic 700/H ultrasonic bath (Singen, Germany).

#### 3.6.1. Identification and Quantification of Polyphenolic Compounds

The previously described LC-QTOF-MS/MS analysis [114,115,116] was used to separate polyphenols on a Zorbax SB-C18 column (100 mm × 3.0 mm i.d., 3.5 µm particle size; Agilent Technologies, Santa Clara, CA, USA) maintained at 48 °C. The mobile phase comprised 0.2% (*v*/*v*) acetic acid in water (solvent A) and 0.2% (*v*/*v*) acetic acid in methanol (solvent B). A gradient elution was applied: 0–30 min, a linear gradient from 3% to 70% B, followed by 4 min of re-equilibration at 5% B. The HPLC parameters were a flow rate of 1 mL/min, a column temperature of 48 °C, and an injection volume of 1 µL. MS detection was performed in negative ionization mode using either selected ion monitoring (SIM) or multiple reaction monitoring (MRM), both with a mass tolerance of 15 ppm [114,115,116]. The source parameters for the heated electrospray ionization (HESI) were source voltage −2000 V, interface temperature 300 °C, nebulizing gas flow 3 L/min, heating gas flow 15 L/min (air), drying gas flow 15 L/min (nitrogen), desolvation line temperature 300 °C, and heat block temperature 400 °C. The QTOF mass spectrometer was operated using multiple-schedule acquisition events for both qualitative and quantitative analysis.

For qualitative analysis, an HR-MS scan time event was scheduled between 0.8 and 30 min, along with a data-dependent acquisition (DDA) over the same time range. The spectral data obtained from the DDA event were used for qualitative confirmation of the compounds (spectral match score > 90 and ion ratio calculation for two qualifier ions from spectra, with an ±20% tolerance compared to library spectral data). In the case of a positive library match, the compound peak area was calculated based on the HR-MS time event, the compound concentration was expressed as equivalents of the specified structurally related reference standard (Table S1).

Quantitative analysis was performed using a specific time-scheduled event for each available standard compound (starting 0.8 min before and ending 0.8 min after its standard retention time) in either HR-MS or HR-MS/MS mode. The quantitation of the compound was done only after positive qualitative identification based on spectral data from DDA events (Table S1).

For compounds quantified using authentic standards, calibration was performed over the concentration range of 0.05–50 µg/mL. Analyte-specific detection thresholds were estimated using a conservative signal-to-noise criterion of at least 10 and ranged from 0.0132 to 0.0256 µg/mL (Table S2). These values are reported as S/N-based detection thresholds rather than conventional limits of detection (LODs). The common lower quantification limit (LOQ) was established at 0.05 µg/mL, the lowest calibration level evaluated for quantitative performance. At this concentration, intra-day precision, inter-day precision, and accuracy were assessed for each analyte; the corresponding results and experimental details are provided in Table S2. Thus, 0.05 µg/mL represents the validated lower quantification limit used in this study and is distinct from the S/N-based detection threshold.

To ensure a maximum mass accuracy of spectral data (<3 ppm mass error), an internal standard calibration time event was run in each analysis between 0.1 and 0.3 min (before the dead time of chromatographic analysis), using a standard solution of sodium iodide at a concentration of 400 mg/L (P/N 220-91239-90, Shimadzu Corporation, Kyoto, Japan). The mass correction was automatically applied to the recorded spectral data by the LabSolutions software version 5.137. Results were expressed as µg/g dw lyophilized extract and as µg/g dw plant material.

#### 3.6.2. Identification and Quantification of Ellagitannins

The chromatographic separation was achieved on a Zorbax SB-C18 column (100 mm × 3.0 mm i.d., 3.5 µm particle size; Agilent Technologies, Santa Clara, CA, USA) maintained at 40 °C. The mobile phase consisted of 0.1% (*v*/*v*) formic acid in water (Solvent A) and acetonitrile (Solvent B). A gradient elution was applied as follows: 0–2 min, linear gradient from 5% to 20% B; followed by a re-equilibration step with 8% B for 3 min. The flow rate was set at 1 mL/min, and the injection volume was 1 µL. Mass-spectrometry detection was performed in negative ionization mode using a HESI ion source. The HESI source parameters were set as follows: source voltage −3000 V, interface temperature 350 °C, nebulizing gas flow 2.5 L/min, heating gas flow 15 L/min (air), drying gas flow 15 L/min (nitrogen), desolvation line temperature 250 °C, and heat block temperature 400 °C. The collision energy was 25 (absolute units) with the ramping option turned off.

The quantitative analysis was performed by monitoring specific fragmentation transitions. For both vescalagin and castalagin (stereoisomers/epimers), the precursor ion at *m*/*z* 466.025 fragmented to yield a product ion at *m*/*z* 300.99, which was used for quantification. Data acquisition and processing were carried out using LabSolutions software version 5.137 (Shimadzu Corporation, Kyoto, Japan).

This LC–MS/MS method was used to identify and quantify the ellagitannins castalagin and vescalagin in WSLE and WPLE. The retention times of vescalagin and castalagin were 1.35 and 1.70 min, respectively, and the total analysis runtime was 2.3 min. The analytical method was validated for linearity, S/N-based detection threshold, lower limit of quantification (LOQ), precision, and accuracy. Calibration for castalagin and vescalagin was performed over 0.05–100 µg/mL. For this dedicated method, detection thresholds were estimated using an S/N criterion of ≥5 (0.0122 µg/mL for castalagin and 0.0171 µg/mL for vescalagin); these values are therefore not directly comparable with the S/N ≥ 10 thresholds reported in Section 3.6.1. The LOQ was validated at 0.05 µg/mL for both analytes. Precision and accuracy results at this concentration were as follows: for castalagin, intra-day precision (*n* = 3) was 7.21%, inter-day precision (*n* = 3) was 9.02%, and accuracy (*n* = 3) was 106.38%. For vescalagin, intra-day precision (*n* = 3) was 5.54%, the inter-day precision (*n* = 3) was 8.83%, and the accuracy (*n* = 3) was 98.54%. The results were expressed as µg/g of lyophilized extract (dw).

### 3.7. Computational Methodology: Molecular Docking and Molecular Dynamics Simulations

#### 3.7.1. Molecular Docking

Ellagic acid, gallic acid, catechin, procyanidin B1, procyanidin B3, and quercitrin, which were identified as the most abundant compounds in the studied extracts, were subjected to a molecular docking study performed using AutoDock Vina 1.2.7 (The Scripps Research Institute, La Jolla, CA, USA) [117] against KEAP1 obtained from the Protein Data Bank (PDB ID: 4L7B) [118,119]. The preparation of the KEAP1 macromolecule comprised the addition of polar hydrogen atoms and assignment of partial atomic charges according to previously reported procedures [120], while the ligands were created using Avogadro software, version 1.2.0 [121]. The final preparation of the ligands in their neutral state and the receptor was completed with AutoDockTools 1.5.6 (The Scripps Research Institute, La Jolla, CA, USA) according to previously reported procedures [120,122].

The search space for molecular docking was centered at x = −4.405, y = 3.384, and z = −28.233, defined as a cube with 20 Å sides. Targeting this pocket is essential because KEAP1 binds Nrf2 here to degrade it, and blocking this interaction allows Nrf2 to stabilize and activate the cell’s antioxidant defense genes. By competitively occupying this site, small molecules can inhibit the protein–protein interaction between KEAP1 and Nrf2, representing a feasible strategy to combat diseases caused by oxidative stress [82,83,123,124]. For clearer results of the binding of the ligands to KEAP1, exhaustiveness was set to 20 and num_modes to 50. The resulting top-binding docking poses of the ligands to KEAP1 were further analyzed and visualized with UCSF Chimera, version 1.16 (Resource for Biocomputing, Visualization, and Informatics, University of California, San Francisco, CA, USA) [125]. The docking protocol was validated by redocking the co-crystallized ligand 1VV, yielding a heavy-atom RMSD of 1.13 Å between the crystallographic and redocked poses, indicating satisfactory reproduction of the experimental binding mode.

#### 3.7.2. Molecular Dynamics Simulation

For each ligand, the top-ranked binding pose to KEAP1, identified by AutoDock Vina in the molecular docking study, was used to build the corresponding complex with the target macromolecule.

Molecular dynamics simulations were subsequently performed using GROMACS version 2023 (KTH Royal Institute of Technology and Stockholm University, Stockholm, Sweden) with the Chemistry at HARvard Macromolecular Mechanics, version 36 (CHARMM36) force field and the transferable intermolecular potential 3-point (TIP3P) water model in an orthorhombic simulation box for 100 ns [126,127]. Ligand parameters were generated using the CHARMM General Force Field (CGenFF) server (University of Maryland School of Pharmacy, Baltimore, MD, USA), and system preparation, including charge neutralization and energy minimization, followed previously reported procedures. To create a realistic physiological environment, the systems were neutralized with Na^+^ and Cl^−^ counterions, and the ionic strength was adjusted to 150 mM NaCl using the GROMACS genion utility. Water molecules were replaced by ions according to the electrostatic potential while maintaining overall charge neutrality [128,129,130].

Energy minimization was performed using the steepest descent algorithm (maximum 50,000 steps, convergence criterion of 1000 kJ mol^−1^ nm^−1^). The minimized systems were subsequently equilibrated for 500 ps under the canonical (NVT) ensemble, followed by 500 ps under the isothermal–isobaric (NPT) ensemble, with position restraints enabled during both equilibration stages. Initial velocities were generated according to the Maxwell–Boltzmann distribution at 300 K. A leap-frog integration algorithm with a timestep of 2 fs was employed, and bonds involving hydrogen atoms were constrained using LINCS. Temperature was maintained at 300 K using the velocity-rescaling (V-rescale) thermostat with a coupling constant of 0.1 ps. During NPT equilibration, pressure was maintained at 1 bar using the C-rescale barostat with isotropic pressure coupling and a coupling constant of 2 ps. The subsequent 100 ns production simulations were performed without the equilibration position restraints, using the Parrinello–Rahman barostat at 1 bar with a coupling constant of 2 ps. Non-bonded interactions were evaluated using the Verlet cutoff scheme, with a cutoff distance of 1.2 nm for both short-range electrostatic and van der Waals interactions. Long-range electrostatic interactions were treated using PME with a grid spacing of 0.16 nm. Three-dimensional periodic boundary conditions were applied along all three spatial directions (x, y, and z).

All computations were carried out on a workstation running Debian Linux 12, equipped with an Intel Core i7-12700KF CPU and an NVIDIA GeForce RTX 3060 GPU, with GPU acceleration provided through CUDA 12.8. Molecular visualization was performed using VMD version 1.9.4 [131]. Trajectory processing and analysis were conducted with the analysis utilities available in GROMACS.

### 3.8. Biological Activities on Cell Lines

#### 3.8.1. Cell Cultures

The intestinal cell line Caco-2 was purchased from CLS Cell Lines Service GmbH (Eppelheim, Germany), and the immune THP-1 and Jurkat cell lines were obtained from American Type Culture Collection (ATCC, Manassas, VA, USA).

The human colon carcinoma Caco-2 cells (ATCC HTB-37) were maintained as a monolayer in 75 cm^2^ polystyrene tissue-culture flasks at a seeding density of 25,000 cells/cm^2^. The complete growth medium consisted of DMEM (4.5 g/L glucose) supplemented with 10% FBS, 1% HEPES, 1% NEAA, and 1% penicillin–streptomycin. The cells were used for the experiments or subcultured once they reached 70–80% confluence.

The Jurkat cells (ATCC TIB-152) and the THP-1 human monocytic cells (ATCC TIB-202) were propagated in RPMI-1640 medium supplemented with 10% FBS and 1% penicillin–streptomycin. Cultures were incubated at 37 °C in a humidified 5% CO_2_ atmosphere and were routinely maintained at 2–8 × 10^5^ cells/mL, and the medium was refreshed every 2 days.

#### 3.8.2. Preparation of Extract Solutions

The stock solutions of the WSLE and WPLE (200 mg/mL) were prepared separately in DMSO. Serial dilutions in DMSO yielded working solutions of 2.5, 5, 12.5, 37.5, 50, 75, 125, and 150 µg/mL. The final concentrations for the in vitro cell line experiments were obtained by diluting these working solutions with cell culture medium.

#### 3.8.3. Cell Viability Assay

The cytotoxicity of the extracts was assessed by the Alamar Blue (resazurin) assay on Caco-2, Jurkat, and THP-1 cells, as previously described [47,132]. The tested concentration ranges for the WSLE and WPLE were 2.5–100 µg/mL in Caco-2 intestinal cells and 2.5–150 µg/mL in the Jurkat and THP-1 immune cell lines. The cells were seeded in 96-well plates (Caco-2 and Jurkat: 60,000 cells/well; THP-1: 30,000 cells/well) and allowed to adhere (Caco-2) or to settle out of suspension (Jurkat and THP-1) for 24 h. After 24 h of exposure, the cells were incubated with 200 µM resazurin for 2–3 h. To assess possible cell-independent interactions between the extracts and resazurin, extract-only controls containing each extract at the tested concentrations and resazurin, but no cells were incubated and measured under the same experimental conditions. Fluorescence was measured on a Synergy 2 Multi-Mode Microplate Reader (λ_excitation_ = 530/25 nm; λ_emission_ = 590/35 nm). The experiments were performed in three independent biological replicates, each comprising six technical replicates, and included a negative control (cells treated with culture medium containing 0.2% (*v*/*v*) DMSO). The results were presented relative to the negative control (100%). Where applicable, the IC_50_ values were derived from dose–response curves by fitting the data to a four-parameter logistic model using SigmaPlot 11.

#### 3.8.4. Cellular Antioxidant Potential

The ability of WSLE and WPLE to reduce oxidative stress in Caco-2 cells was assessed using the ROS-sensitive probe 2′,7′-dichlorodihydrofluorescein diacetate (DCFH-DA) [133]. Caco-2 cells were selected for their retained active membrane transport processes, which make them a physiologically relevant model for assessing the antioxidant activity of dietary phenolic compounds. The cells were treated for 24 h with three non-cytotoxic concentrations of each extract (2.5, 7.5, and 12.5 µg/mL). After treatment, the cells were washed with PBS and loaded with 50 µM DCFH-DA in Hanks’ Balanced Salt Solution (HBSS) for 2 h. Excess dye was removed by washing, and the cells were then incubated for a further 2 h with either 250 µM H_2_O_2_ (stimulated condition) or HBSS (basal condition) to quantify ROS production. Oxidation of 2′,7′-dichlorodihydrofluorescein (DCFH), a metabolite formed intracellularly by esterase activity, to the fluorescent product 2′,7′-dichlorofluorescein (DCF) was measured on a Synergy 2 Multi-Mode Microplate Reader (λ_excitation_ = 485/20 nm; λ_emission_ = 528/20 nm). A 5 mM concentration of NAC was used as a reference antioxidant.

#### 3.8.5. Cellular Anti-Inflammatory Potential

The anti-inflammatory potential of WSLE and WPLE was evaluated by quantifying the pro-inflammatory cytokines IL-1β, IL-6, IL-8, and TNF-α in the culture supernatants of Caco-2, Jurkat, and THP-1 cells. Preliminary experiments were performed to select a responsive cell model and biomarker. Caco-2, Jurkat, and THP-1 cells were exposed to increasing concentrations of LPS from *E. coli* (Sigma-Aldrich, Steinheim, Germany), up to 100 ng/mL, for 24 h. IL-1β, IL-6, IL-8, and TNF-α levels were then measured in the culture supernatants as described below. The cell line and biomarker used in the subsequent experiments were selected based on the magnitude and statistical significance of the LPS-induced response compared with unstimulated controls.

For the anti-inflammatory assay, THP-1 cells were incubated for 24 h with 100 ng/mL LPS in the presence of three non-cytotoxic concentrations of WSLE and WPLE (5, 25, and 50 µg/mL), selected based on the cytocompatibility results (Section 3.8.3). LPS-stimulated cells without extract and unstimulated cells served as positive and negative controls, respectively. Before assessing anti-inflammatory effects, cell viability was determined to exclude additional cytotoxicity of the extracts in the presence of LPS. After 24 h of treatment, culture supernatants were collected and stored at −70 °C until analysis.

Cytokine concentrations were quantified using commercial ELISA kits (FineTest Biotech Inc., Wuhan, China), according to the manufacturer’s instructions. The kits used were Human IL-1β (Cat. No. EH0185), Human IL-6 (Cat. No. EH0201), Human IL-8 (Cat. No. EH0205), and Human TNF-α (Cat. No. EH0302). A cytokine standard curve was included in each assay, and cytokine concentrations were calculated from it [134].

### 3.9. Statistical Analysis

Data acquisition and processing for the phytochemical analysis of bioactive compounds by LC-HRMS were performed using LabSolutions software version 5.137 (Shimadzu Corporation, Kyoto, Japan). Three independent extracts (*n* = 3) were prepared in parallel from each matrix. For phytochemical analyses, each extract was analyzed in technical triplicate. Cell-based assays were conducted in three independent experiments, each using a separate extract and six technical replicates for each experimental group. Technical replicates were averaged within each experiment to yield one value for each experimental group (*n* = 3). For the cell viability and cellular antioxidant assays, values from each independent experiment were normalized to the negative control measured on the same day.

The results were reported as mean ± SD. Statistical analysis was performed using SigmaPlot 11.0 software. Normality (Shapiro–Wilk test) and homogeneity of variances were checked; however, given the very limited power of these tests with *n* = 3, they were used only as a screening tool rather than as confirmation of normality. Phytochemical data and normalized cell-based data (cell viability and cellular antioxidant activity) were analyzed by one-way analysis of variance (ANOVA). Because IL-8 data were expressed as absolute concentrations, not normalized to the daily control, and all treatment groups were included in each independent experiment, IL-8 data were analyzed by repeated-measures ANOVA, with the independent experiment as the blocking factor. The Holm–Šídák post hoc test was applied for multiple comparisons. A probability value (*p*) of less than 0.05 was considered statistically significant.

## 4. Conclusions

This study compared two *Juglans regia* L. processing by-products, walnut septum and pellicle, by combining targeted phytochemical profiling with chemical, computational, and cell-based evaluations of their biological properties. The two matrices showed distinct polyphenolic profiles. The pellicle had a higher total phenolic content and was richer in ellagic acid, gallic acid, and castalagin, whereas the septum contained higher levels of condensed tannins, catechin, procyanidins, and quercitrin. Consistent with its higher phenolic content, the pellicle extract generally showed greater antioxidant capacity in the ABTS, DPPH, and FRAP assays. Nevertheless, both extracts attenuated the H_2_O_2_-induced increase in intracellular DCF fluorescence in Caco-2 cells at non-cytotoxic concentrations, suggesting that their cellular antioxidant effects cannot be inferred solely from their chemical radical-scavenging capacity.

Molecular docking and molecular dynamics simulations identified several major phenolic constituents as potential KEAP1 ligands, with quercitrin and catechin predicted to form the most stable complexes. These in silico findings are hypothesis-generating, and experimental validation is required before establishing any involvement of the KEAP1/Nrf2 pathway in the antioxidant activities of the extracts. Under the tested conditions, LPS induced a pro-inflammatory response only in THP-1 cells. In these cells, both extracts reduced LPS-induced IL-8 secretion, which provides preliminary evidence of anti-inflammatory potential.

Overall, walnut septum and pellicle are chemically distinct sources of polyphenols with measurable antioxidant activity and preliminary cell-based bioactivity, supporting their valorization as food-processing by-products. Future research should include additional inflammatory endpoints, differentiated intestinal barrier models, epithelial–immune co-culture systems, isolated compounds, polyphenol-derived metabolites, and, subsequently, in vivo and clinical studies to elucidate the underlying mechanisms of action and establish their translational relevance.

## Figures and Tables

**Table 1:** Quantitative analysis of total bioactive compounds in the walnut septum and pellicle extracts.

Plant Matrices	TPC (mg GAE/g dw)	TFC (mg QE/g dw)	CTC (mg CE/g dw)
WS	107.83 ± 0.13 ^a^	9.71 ± 0.23 ^a^	286.85 ± 6.79 ^a^
WP	218.09 ± 0.91 ^b^	9.13 ± 0.09 ^a^	97.02 ± 2.62 ^b^

CE—catechin equivalents; CTC—condensed tannin content; GAE—gallic acid equivalents; TFC—total flavonoid content; TPC—total phenolic content; QE—quercetin equivalents; WP—walnut pellicle; WS—walnut septum. Data are presented as mean ± SD of three independent extraction replicates (*n* = 3) and are expressed on a dry-weight (dw) plant material basis. Different superscript letters indicate statistically significant differences between extracts (one-way analysis of variance (ANOVA) followed by Holm–Šídák multiple comparisons test, *p* < 0.05).

**Table 2:** Antioxidant activities of walnut septum and pellicle extracts determined by ABTS, DPPH, and FRAP assays.

Plant Matrices	ABTS-TEAC (mg TE/g dw)	DPPH (mg TE/g dw)	FRAP (mg TE/g dw)
WS	210.40 ± 6.97 ^a^	235.94 ± 3.76 ^a^	660.80 ± 7.79 ^a^
WP	428.14 ± 6.44 ^b^	548.21 ± 3.04 ^b^	916.36 ± 2.41 ^b^

ABTS—2,2’-azino-bis(3-ethylbenzothiazoline-6-sulfonic acid); DPPH—2,2-diphenyl-1-picrylhydrazyl; FRAP—ferric reducing antioxidant power; TE—Trolox equivalents; TEAC—Trolox equivalent antioxidant capacity; WP—walnut pellicle; WS—walnut septum. Data are presented as mean ± SD of three independent extraction replicates (*n* = 3) and are expressed on a dry-weight (dw) plant material basis. Different superscript letters indicate statistically significant differences between extracts (one-way ANOVA followed by Holm–Šídák multiple comparisons test, *p* < 0.05).

**Table 3:** Polyphenolic compounds identified and quantified in lyophilized walnut septum and pellicle extracts by LC–MS/MS.

Classification		Compounds	Detection Mode	Amount	Amount	Amount	Amount
				(µg/g dw WSLE)	(µg/g dw WS *)	(µg/g dw WPLE)	(µg/g dw WP *)
Phenolic acids	Hydroxy-benzoic acids	**Ellagic acid**	HR-SIM	**5228.94 ± 423.54 ^a^**	492.14 ± 39.86 ^A^	**8377.06 ± 678.54 ^b^**	2033.12 ± 164.68 ^B^
		**Gallic acid**	HR-MRM	**431.29 ± 36.23 ^a^**	40.60 ± 3.41 ^A^	**706.01 ± 59.31 ^b^**	171.34 ± 14.40 ^B^
		Protocatechuic acid	HR-MRM	157.27 ± 15.10 ^a^	14.80 ± 1.42 ^A^	14.54 ± 1.40 ^b^	3.53 ± 0.34 ^B^
		Syringic acid	HR-MRM	86.08 ± 6.11	8.11 ± 0.58	ND	ND
		Vanillic acid	HR-MRM	122.84 ± 11.92	11.56 ± 1.12	ND	ND
	Hydroxy-cinnamic acids	Caffeic acid	HR-MRM	7.83 ± 0.31	0.73 ± 0.03	<LOQ	<LOQ
		4-*O*-Caffeoyl-quinic acid	HR-MRM	39.57 ± 2.02	3.73 ± 0.19	ND	ND
		Chlorogenic acid	HR-MRM	9.58 ± 0.44 ^a^	0.90 ± 0.04 ^A^	11.63 ± 0.54 ^b^	2.82 ± 0.13 ^B^
		*p*-Coumaric acid	HR-MRM	14.470 ± 0.64 ^a^	1.36 ± 0.06 ^A^	6.06 ± 0.27 ^b^	1.47 ± 0.07 ^A^
Flavonoids	Flavan-3-ols	**Catechin**	HR-SIM	**3989.51 ± 147.61 ^a^**	375.49 ± 13.89 ^A^	**1447.80 ± 53.57 ^b^**	351.38 ± 13.00 ^B^
		Epicatechin	HR-SIM	55.24 ± 1.71	5.20 ± 0.16	ND	ND
		Gallocatechin	HR-MRM	10.03 ± 0.45	0.94 ± 0.04	ND	ND
		**Procyanidin B1**	HR-MRM	**2082.28 ± 106.18** **^a^**	195.98 ± 9.99 ^A^	**392.11 ± 19.89 ^b^**	95.17 ± 4.83 ^B^
		Procyanidin B2	HR-MRM	ND	ND	27.01 ± 2.00	6.56 ± 0.48
		**Procyanidin B3**	HR-MRM	**2785.61 ± 161.57 ^a^**	262.18 ± 15.20 ^A^	**663.27 ± 38.47 ^b^**	160.98 ± 9.34 ^B^
		Procyanidin B4	HR-MRM	235.05 ± 18.33 ^a^	22.12 ± 1.73 ^A^	26.70 ± 2.08 ^b^	6.48 ± 0.50 ^B^
	Flavonols	Quercetin	HR-MRM	41.71 ± 2.92	3.93 ± 0.27	ND	ND
		Hyperoside	HR-MRM	85.03 ± 7.99 ^a^	8.00 ± 0.75 ^A^	9.32 ± 0.88 ^b^	2.26 ± 0.21 ^B^
		Isoquercitrin	HR-MRM	310.56 ± 30.12 ^a^	29.23 ± 2.83 ^A^	20.270 ± 1.97 ^b^	4.93 ± 0.48 ^B^
		**Quercitrin**	HR-MRM	**3112.64 ± 127.62**	292.96 ± 12.02	ND	ND
		Quercetin-3-*O*-glucose-6-acetate	HR-MRM	38.84 ± 3.15	3.66 ± 0.30	ND	ND
	Flavones	Acacetin	HR-MRM	<LOQ	<LOQ	ND	ND
		Apigenin	HR-SIM	9.12 ± 0.68	0.86 ± 0.06	ND	ND
Phenyl-propanoids	Coumarins	Esculetin	HR-SIM	<LOQ	<LOQ	ND	ND
		Scopoletin	HR-SIM	10.85 ± 0.89	1.02 ± 0.08	ND	ND
		Umbelliferone	HR-SIM	14.19 ± 0.82	1.33 ± 0.08	ND	ND
	Phenylpropanoid aldehydes	Coniferyl aldehyde	HR-MRM	12.24 ± 0.99	1.15 ± 0.09	ND	ND
		Sinapaldehyde	HR-MRM	10.85 ± 0.38	1.02 ± 0.04	ND	ND
Hydrolysable tannins	Ellagitannins	**Castalagin**	HR-MRM	ND	ND	**591.81 ± 33.14**	143.63 ± 8.04
		Vescalagin	HR-MRM	ND	ND	ND	ND

LOQ—limit of quantification; HR-MRM—high-resolution multiple reaction monitoring; ND—not detected; HR-SIM—high-resolution selected ion monitoring; WPLE—walnut pellicle lyophilized extract; WSLE—walnut septum lyophilized extract. *—WP (walnut pellicle) and WS (walnut septum) refer to the raw plant material; their associated values are calculated from the initial quantity used for extraction. Data are presented as mean values ± SD of three independent extraction replicates (*n* = 3). Different letters (a, b for the lyophilized extracts and A, B for the raw plant materials) indicate statistically significant differences between the septum and pellicle (ANOVA + Holm–Šídák post hoc test at *p* < 0.05). Major compounds discussed in the text are highlighted in bold.

**Table 4:** The binding affinity (ΔG) of ellagic acid, gallic acid, catechin, procyanidin B1, procyanidin B3, and quercitrin to KEAP1.

Compound	ΔG (kcal/mol)
ellagic acid	−8.4
gallic acid	−6.3
catechin	−7.9
procyanidin B1	−8.7
procyanidin B3	−8.0
quercitrin	−9.4

ΔG—Predicted binding affinity (kcal/mol); KEAP1—Kelch-like ECH-associated protein 1; more negative values indicate stronger binding. Molecular docking was performed using AutoDock Vina 1.2.7 against KEAP1 (PDB ID: 4L7B).

**Table 5:** Analysis of the trajectories of the systems during molecular dynamics simulations.

System Evaluated	RMSD Ligand (nm)	Ligand Behavior from the Initial Binding Pose			KEAP1-Ligand Hydrogen Bonds (Mean)	RMSF Amino Acid αCarbon Atoms (nm)	RMSF Amino Acid Side Chains (nm)	RMSD Protein Backbone (nm)	Rg Protein (nm)
		Qualitative Assessment	Distance ^1^ (nm)	Maximum Distance ^1^ (nm)					
KEAP1 + ellagic acid	0.57 ± 0.15	moderate deviation	0.48 ± 0.08	0.91	1.07 ± 0.89	0.07 ± 0.08	0.12 ± 0.09	0.14 ± 0.04	1.80 ± 0.01
KEAP1 + gallic acid	1.21 ± 0.84	substantial deviation	1.69 ± 0.75	4.66	-	−0.08 ± 0.07	−0.12 ± 0.09	−0.18 ± 0.02	−1.80 ± 0.01
KEAP1 + catechin	0.36 ± 0.04	limited deviation	0.55 ± 0.05	0.72	1.46 ± 0.66	0.08 ± 0.08	0.13 ± 0.11	0.18 ± 0.04	1.81 ± 0.01
KEAP1 + procyanidin B1	0.68 ± 0.14	moderate deviation	0.82 ± 0.13	1.64	1.92 ± 0.97	0.08 ± 0.09	0.13 ± 0.11	0.16 ± 0.02	1.81 ± 0.01
KEAP1 + procyanidin B3	1.30 ± 0.84	substantial deviation	1.31 ± 0.57	3.00	-	−0.07 ± 0.08	−0.12 ± 0.10	−0.17 ± 0.04	−1.80 ± 0.01
KEAP1 + quercitrin	0.24 ± 0.05	limited deviation	0.43 ± 0.03	0.53	2.61 ± 1.25	0.06 ± 0.06	0.11 ± 0.06	0.15 ± 0.01	1.81 ± 0.01
KEAP1	N/A	N/A			N/A	0.07 ± 0.07	0.11 ± 0.09	0.17 ± 0.04	1.81 ± 0.01

KEAP1—Kelch-like ECH-associated protein 1; N/A—not applicable; Rg—radius of gyration; RMSD—root mean square deviation; ^1^ The distance was calculated between the ligand center of mass and the geometric center defined by the αC atoms of selected KEAP1 binding-site residues Arg380, Asn382, Arg415, Arg483, Ser508, and Ser555; RMSD protein backbone—overall structural stability of KEAP1; RMSF—root mean square fluctuation; (-) indicates parameters not calculated due to dissociation. Data are presented as mean values ± SD.

**Table 6:** IC_50_ values (µg/mL) after exposure of Jurkat, Caco-2, and THP-1 cells to walnut septum lyophilized extract (WSLE) and walnut pellicle lyophilized extract (WPLE), evaluated by the Alamar Blue assay. Results are expressed as means ± SD (*n* = 3).

Cell Line	WSLE	WPLE
Caco-2 cell line	28.21 ± 0.72	>100
Jurkat cell line	74.38 ± 1.96	118.95 ± 2.48
THP-1 cell line	>150	>150

## Data Availability

The original contributions presented in this study are included in the article/Supplementary Materials. Further inquiries can be directed to the corresponding author.

## Supplementary Materials

The following supporting information can be downloaded at https://www.mdpi.com/article/10.3390/ph19101657/s1, Table S1: Phenolic compounds assessed by qualitative analysis (authentic standards) using HR-MS or HR-MS/MS—chromatographic and detection data; Table S2: Calibration parameters, S/N-based detection thresholds and quantitative performance at 0.05 µg/mL for phenolic compounds quantified using authentic standards; Figure S1. Effect of lipopolysaccharide (LPS) on cell viability.

## Abbreviations

The following abbreviations are used in this manuscript: AA—antioxidant activity; ABTS—2,2′-azino-bis(3-ethylbenzothiazoline-6-sulfonic acid); ARE—antioxidant response element; CE—catechin equivalents; CTC—condensed tannin content; CUDA—compute unified device architecture; DCF—2′,7′-dichlorofluorescein; DCFH-DA—2′,7′-dichlorodihydrofluorescein diacetate; DMEM—Dulbecco’s modified Eagle’s medium; DMSO—dimethyl sulfoxide; DPPH—2,2-diphenyl-1-picrylhydrazyl; dw—dry weight; ΔG—predicted binding affinity; EA—ellagic acid; EAE—enzyme-assisted extraction; ESI—electrospray ionization; FBS—fetal bovine serum; FRAP—ferric reducing antioxidant power; GAE—gallic acid equivalents; GA—gallic acid; GROMACS—GROningen MAchine for chemical simulations; HR-MRM—high-resolution multiple reaction monitoring; HR-SIM – high-resolution selected ion monitoring; HO-1—heme oxygenase-1; HPLC—high-performance liquid chromatography; HRMS—high-resolution mass spectrometry; IL—interleukin; KEAP1—Kelch-like ECH-associated protein 1; LC—liquid chromatography; LOD—limit of detection; LOQ—limit of quantification; LPS—lipopolysaccharides; MD—molecular dynamics; ME—methanolic condensation reflux extraction; MS—mass spectrometry; NAC—N-acetylcysteine; ND—not detected; NEAA—non-essential amino acids; NF-κB—nuclear factor kappa-light-chain-enhancer of activated B cells; Nrf2—nuclear factor erythroid 2-related factor 2; PDB—protein data bank; PBS—phosphate-buffered saline; QE—quercetin equivalents; QTOF—quadrupole time-of-flight; RMSD—root mean square deviation; RMSF—root mean square fluctuation; RNS—reactive nitrogen species; RPMI—Roswell Park Memorial Institute medium; ROS—reactive oxygen species; SD—standard deviation; TAA—total antioxidant activity; TE—Trolox equivalents; TEAC—Trolox equivalent antioxidant capacity; TFC—total flavonoid content; TNF-α—tumor necrosis factor alpha; TPC—total phenolic content; TPTZ—2,4,6-tris(2-pyridyl)-s-triazine; Trolox—6-hydroxy-2,5,7,8-tetramethylchroman-2-carboxylic acid; UAE—ultrasonic-assisted extraction; UPLC—ultra-performance liquid chromatography; UTE—Ultra-Turrax extraction; VMD—visual molecular dynamics; WP—walnut pellicle; WPE—walnut pellicle extract; WPLE—walnut pellicle lyophilized extract; WS—walnut septum; WSE—walnut septum extract; WSLE—walnut septum lyophilized extract.
