# Supplementary material for: Comparative Phytochemical and Biological Evaluation of Walnut Septum and Pellicle Extracts in Chemical, Computational, and Cell-Based Models
Source: Pharmaceuticals (Basel). 2026 Oct 20;19(10):1657. doi: 10.3390/ph19101657 (PMC13708377; doi:10.3390/ph19101657)
Supplement: Supplementary file 1 [file pharmaceuticals-19-01657-s001.zip › pharmaceuticals-4591814-supplementary.pdf]

pharmaceuticals

Supplementary file

Comparative Phytochemical and Biological Evaluation of Walnut Septum and Pellicle Extracts in Chemical, Computational, and Cell-Based Models

Letiția Mateș [1,†], Denisia Pașca [2,†], Ionel Fizeșan [1], Ana-Maria Vlase [3,*], Gabriel Marc [4], Ionuț-Valentin Creștin [1], Oana Andreea Alexa [1], Ioan Tomuță [5], Lorena Filip [6,7], Laurian Vlase [5] and Daniela-Saveta Popa [1]

[1] Department of Toxicology, Faculty of Pharmacy, Iuliu Hatieganu University of Medicine and Pharmacy, 8 Victor Babeș, 400012 Cluj-Napoca, Romania; micu.letitia@umfcluj.ro (L.M.); ionel.fizesan@umfcluj.ro (I.F.); ionut.vale.crestin@elearn.umfcluj.ro (I.-V.C.); alexaoana9@gmail.com (O.A.A.); dpopa@umfcluj.ro (D.-S.P.)
[2] Department of Nutrition and Medical Cosmetics, Faculty of Nursing and Health Sciences, Iuliu Hatieganu University of Medicine and Pharmacy, 23 Gheorghe Marinescu, 400337 Cluj-Napoca, Romania; denisia.pasca@umfcluj.ro
[3] Department of Pharmaceutical Botany, Faculty of Pharmacy, Iuliu Hatieganu University of Medicine and Pharmacy, 8 Victor Babeș, 400012 Cluj-Napoca, Romania
[4] Department of Organic Chemistry, Faculty of Pharmacy, Iuliu Hatieganu University of Medicine and Pharmacy, 8 Victor Babeș, 400012 Cluj-Napoca, Romania; marc.gabriel@umfcluj.ro
[5] Department of Pharmaceutical Technology and Biopharmacy, Faculty of Pharmacy, Iuliu Hatieganu University of Medicine and Pharmacy, 8 Victor Babes, 400012 Cluj-Napoca, Romania; tomutaioan@umfcluj.ro (I.T.); laurian.vlase@umfcluj.ro (L.V.)
[6] Department of Bromatology, Hygiene, Nutrition, Faculty of Pharmacy, Iuliu Hatieganu University of Medicine and Pharmacy, 6 Louis Pasteur, 400349 Cluj-Napoca, Romania; lfilip@umfcluj.ro
[7] Academy of Romanian Scientists (AOSR), 3 Ilfov Street, 050044 Bucharest, Romania
* Correspondence: gheldiu.ana@umfcluj.ro
† These authors contributed equally to this work.

Academic Editor: Grażyna Zgórka

Received: 12 September 2026
Revised: 13 October 2026
Accepted: 16 October 2026
Published: 20 October 2026

Copyright: © 2026 by the authors. Licensee MDPI, Basel, Switzerland. This article is an open access article distributed under the terms and conditions of the Creative Commons Attribution (CC BY) license.

Table S1. Phenolic compounds assessed by qualitative analysis (authentic standards) using HR-MS or HR-MS/MS – chromatographic and detection.

No.	Compound name	Molecular formula	Retention time, min	MS analysis type	Precursor ion, *m/z*	Measured ion *m/z*	MS/MS qualifier ion 1 *m/z*	MS/MS qualifier ion 1 formula	MS/MS qualifier ion 2 *m/z*	MS/MS qualifier ion 2 formula	Qualifier 1:2 ratio
1	Gallic acid	$C_7H_6O_5$	1.36	HR-MRM	169.0142	125.0244	125.0244	$C_6H_5O_3^-$	79.0189	$C_5H_3O_1^-$	100 / 40
2	Protocatechuic acid	$C_7H_6O_4$	2.67	HR-MRM	153.0193	109.0295	109.0295	$C_6H_5O_2^-$	91.0189	$C_6H_3O_1^-$	100 / 26
3	Gentisic acid	$C_7H_6O_4$	3.30	HR-MRM	153.0193	109.0295	109.0295	$C_6H_5O_2^-$	108.0217	$C_6H_4O_2^-$	100 / 45
4	Gallocatechin	$C_{15}H_{14}O_7$	2.84	HR-MRM	305.0667	125.0244	179.0350	$C_9H_7O_4^-$	125.0244	$C_6H_5O_3^-$	100 / 60
5	Epigallocatechin	$C_{15}H_{14}O_7$	3.10	HR-MRM	305.0667	125.0244	179.0350	$C_9H_7O_4^-$	125.0244	$C_6H_5O_3^-$	100 / 58
6	Salicin	$C_{13}H_{18}O_7$	4.42	HR-SIM	285.0980	285.0980	123.0452	$C_7H_7O_2^-$	105.0346	$C_7H_5O_1^-$	100 / 31
7	Procyanidin B3	$C_{30}H_{26}O_{12}$	4.75	HR-MRM	577.1351	289.0718	289.0718	$C_{15}H_{13}O_6^-$	425.0878	$C_{22}H_{17}O_9^-$	100 / 45
8	Procyanidin B1	$C_{30}H_{26}O_{12}$	5.20	HR-MRM	577.1351	289.0718	289.0718	$C_{15}H_{13}O_6^-$	425.0878	$C_{22}H_{17}O_9^-$	100 / 38
9	Procyanidin B4	$C_{30}H_{26}O_{12}$	6.15	HR-MRM	577.1351	289.0718	289.0718	$C_{15}H_{13}O_6^-$	425.0878	$C_{22}H_{17}O_9^-$	100 / 40
10	Procyanidin B2	$C_{30}H_{26}O_{12}$	7.40	HR-MRM	577.1351	289.0718	289.0718	$C_{15}H_{13}O_6^-$	425.0878	$C_{22}H_{17}O_9^-$	100 / 46
11	3-Hydroxybenzoic acid	$C_7H_6O_3$	5.72	HR-MRM	137.0244	93.0346	93.0346	$C_6H_5O_1^-$	65.0397	$C_5H_5^-$	100 / 26
12	Esculin	$C_{15}H_{16}O_9$	5.76	HR-MRM	339.0722	177.0193	177.0193	$C_9H_5O_4^-$	133.0295	$C_8H_5O_2^-$	100 / 32
13	Catechin	$C_{15}H_{14}O_6$	5.81	HR-SIM	289.0718	289.0718	245.0819	$C_{14}H_{13}O_4^-$	205.0506	$C_{11}H_9O_4^-$	100 / 63
14	Vanillic acid	$C_8H_8O_4$	6.39	HR-MRM	167.0350	152.0115	152.0115	$C_7H_4O_4^-$	123.0452	$C_7H_7O_2^-$	100 / 80
15	Chlorogenic acid	$C_{16}H_{18}O_9$	6.45	HR-MRM	353.0878	191.0561	191.0561	$C_7H_{11}O_6^-$	179.0350	$C_9H_7O_4^-$	100 / 8
16	Caffeic acid	$C_9H_8O_4$	6.62	HR-MRM	179.0350	135.0452	135.0452	$C_8H_7O_2^-$	179.0350	$C_9H_7O_4^-$	100 / 22
17	cis-Caffeic acid	$C_9H_8O_4$	6.75	HR-MRM	179.0350	135.0452	135.0452	$C_8H_7O_2^-$	179.0350	$C_9H_7O_4^-$	100 / 24
18	Esculetin	$C_9H_6O_4$	6.68	HR-SIM	177.0193	177.0193	133.0295	$C_8H_5O_2^-$	89.0397	$C_7H_5^-$	100 / 50
19	4-*O*-Caffeoylquinic acid	$C_{16}H_{18}O_9$	7.35	HR-MRM	353.0878	173.0455	173.0455	$C_7H_9O_5^-$	191.0561	$C_7H_{11}O_6^-$	100 / 65
20	Syringic acid	$C_9H_{10}O_5$	8.08	HR-MRM	197.0455	182.0221	182.0221	$C_8H_6O_5^-$	153.0557	$C_8H_9O_3^-$	100 / 31
21	Epigallocatechin gallate	$C_{22}H_{18}O_{11}$	8.31	HR-MRM	457.0776	169.0142	169.0142	$C_7H_5O_5^-$	305.0667	$C_{15}H_{13}O_7^-$	100 / 40
22	Epicatechin	$C_{15}H_{14}O_6$	8.89	HR-SIM	289.0718	289.0718	245.0819	$C_{14}H_{13}O_4^-$	205.0506	$C_{11}H_9O_4^-$	100 / 62
23	*p*-Coumaric acid	$C_9H_8O_3$	9.20	HR-MRM	163.0401	119.0502	119.0502	$C_8H_7O_1^-$	163.0401	$C_9H_7O_3^-$	100 / 16
24	*cis-p*-Coumaric acid	$C_9H_8O_3$	9.50	HR-MRM	163.0401	119.0502	119.0502	$C_8H_7O_1^-$	163.0401	$C_9H_7O_3^-$	100 / 22
25	Procyanidin C1	$C_{45}H_{38}O_{18}$	9.35	HR-SIM	865.1985	865.1985	577.1351	$C_{30}H_{25}O_{12}^-$	289.0718	$C_{15}H_{13}O_6^-$	100 / 76
26	Genistin	$C_{21}H_{20}O_{10}$	9.40	HR-MRM	431.0984	269.0455	269.0455	$C_{15}H_9O_5^-$	268.0377	$C_{15}H_8O_5^-$	100 / 13
27	Umbeliferone	$C_9H_6O_3$	9.60	HR-SIM	161.0244	161.0244	117.0346	$C_8H_5O_1^-$	161.0244	$C_9H_5O_3^-$	100 / 15

https://doi.org/10.3390/ph19101657

No.	Compound name	Molecular formula	Retention time, min	MS analysis type	Precursor ion, *m/z*	Measured ion *m/z*	MS/MS qualifier ion 1 *m/z*	MS/MS qualifier ion 1 formula	MS/MS qualifier ion 2 *m/z*	MS/MS qualifier ion 2 formula	Qualifier 1:2 ratio
28	Procyanidin A1	$C_{30}H_{24}O_{12}$	9.70	HR-SIM	575.1195	575.1195	423.0722	$C_{22}H_{15}O_9^-$	289.0718	$C_{15}H_{13}O_6^-$	100 / 61
29	Syringaldehyde	$C_9H_{10}O_4$	9.71	HR-MRM	181.0506	151.0031	166.0266	$C_8H_6O_4^-$	151.0031	$C_7H_3O_4^-$	100 / 45
30	Salicylic acid	$C_7H_6O_3$	10.28	HR-MRM	137.0244	93.0346	93.0346	$C_6H_5O_1^-$	65.0397	$C_5H_5^-$	100 / 15
31	Scopoletin	$C_{10}H_8O_4$	10.80	HR-SIM	191.0350	191.0350	176.0115	$C_9H_4O_4^-$	148.0166	$C_8H_4O_3^-$	100 / 77
32	Ferulic acid	$C_{10}H_{10}O_4$	11.05	HR-MRM	193.0506	134.0373	149.0608	$C_9H_9O_2^-$	134.0373	$C_8H_6O_2^-$	100 / 60
33	*cis*-Ferulic acid	$C_{10}H_{10}O_4$	11.55	HR-MRM	193.0506	134.0373	149.0608	$C_9H_9O_2^-$	134.0373	$C_8H_6O_2^-$	100 / 63
34	Procyanidin A2	$C_{30}H_{24}O_{12}$	12.20	HR-SIM	575.1195	575.1195	423.0722	$C_{22}H_{15}O_9^-$	289.0718	$C_{15}H_{13}O_6^-$	100 / 58
35	Coniferyl aldehyde	$C_{10}H_{10}O_3$	12.25	HR-MRM	177.0557	162.0322	162.0322	$C_9H_6O_3^-$	149.0608	$C_9H_9O_2^-$	100 / 50
36	*o*-Coumaric acid	$C_9H_8O_3$	13.00	HR-MRM	163.0401	119.0502	119.0502	$C_8H_7O_1^-$	163.0401	$C_9H_7O_3^-$	100 / 22
37	Sinapaldehyde	$C_{11}H_{12}O_4$	13.30	HR-MRM	207.0663	177.0193	192.0428	$C_{10}H_8O_4^-$	177.0193	$C_9H_5O_4^-$	100 / 56
38	Vitexin	$C_{21}H_{20}O_{10}$	13.95	HR-MRM	431.0984	311.0561	311.0561	$C_{17}H_{11}O_6^-$	283.0612	$C_{16}H_{11}O_5^-$	100 / 60
39	Isovitexin	$C_{21}H_{20}O_{10}$	14.93	HR-MRM	431.0984	311.0561	311.0561	$C_{17}H_{11}O_6^-$	283.0612	$C_{16}H_{11}O_5^-$	100 / 60
40	Vitexin-2-*O*-rhamnoside	$C_{27}H_{30}O_{14}$	14.50	HR-MRM	577.1563	293.0455	431.0984	$C_{15}H_{17}O_6^-$	311.0561	$C_{17}H_{11}O_6^-$	100 / 80
41	Hyperoside	$C_{21}H_{20}O_{12}$	14.90	HR-MRM	463.0882	300.0276	300.0276	$C_{15}H_8O_7^-$	301.0354	$C_{15}H_9O_7^-$	100 / 81
42	Isoquercitrin	$C_{21}H_{20}O_{12}$	15.35	HR-MRM	463.0882	300.0276	300.0276	$C_{15}H_8O_7^-$	301.0354	$C_{15}H_9O_7^-$	100 / 85
43	*trans*-Resveratrol	$C_{14}H_{12}O_3$	15.10	HR-MRM	227.0714	143.0502	185.0608	$C_{12}H_9O_2^-$	143.0502	$C_{10}H_7O_1^-$	100 / 32
44	Quercetin-3-*O*-robinobioside	$C_{27}H_{30}O_{16}$	15.15	HR-MRM	609.1461	300.0276	301.0354	$C_{15}H_9O_7^-$	300.0276	$C_{15}H_8O_7^-$	100 / 72
45	Rutin	$C_{27}H_{30}O_{16}$	15.50	HR-MRM	609.1461	300.0276	301.0354	$C_{15}H_9O_7^-$	300.0276	$C_{15}H_8O_7^-$	100 / 90
46	Ellagic acid	$C_{14}H_6O_8$	15.60	HR-SIM	300.9990	300.9990	257.0092	$C_{13}H_5O_6^-$	229.0142	$C_{12}H_5O_5^-$	100 / 43
47	Rosmarinic acid	$C_{18}H_{16}O_8$	16.10	HR-MRM	359.0772	161.0244	161.0244	$C_9H_5O_3^-$	197.0455	$C_9H_9O_5^-$	100 / 45
48	Myricetin	$C_{15}H_{10}O_8$	16.30	HR-SIM	317.0303	317.0303	178.9986	$C_8H_3O_5^-$	151.0037	$C_7H_3O_4^-$	100 / 82
49	Hesperidin	$C_{28}H_{34}O_{15}$	16.70	HR-MRM	609.1825	301.0718	301.0718	$C_{16}H_{13}O_6^-$	286.0483	$C_{15}H_{10}O_6^-$	100 / 30
50	Apigenin 7-glucoside	$C_{21}H_{20}O_{10}$	17.00	HR-MRM	431.0984	268.0377	269.0455	$C_{15}H_8O_5^-$	117.0346	$C_8H_5O_1^-$	100 / 12
51	Quercitrin	$C_{21}H_{20}O_{11}$	17.11	HR-MRM	447.0933	300.0276	301.0354	$C_{15}H_9O_7^-$	300.0276	$C_{15}H_8O_7^-$	100 / 86
52	Fisetin	$C_{15}H_{10}O_6$	17.30	HR-SIM	285.0405	285.0405	135.0452	$C_8H_7O_2^-$	121.0295	$C_7H_5O_2^-$	100 / 50
53	Kaempferol-3-*O*-glucoside	$C_{21}H_{20}O_{11}$	17.39	HR-MRM	447.0933	284.0326	284.0326	$C_{15}H_8O_6^-$	285.0405	$C_{15}H_9O_6^-$	100 / 41
54	Quercetin-3-*O*-glucose-6-acetate	$C_{23}H_{22}O_{13}$	17.40	HR-MRM	505.0988	300.0276	301.0354	$C_{15}H_9O_7^-$	300.0276	$C_{15}H_8O_7^-$	100 / 90
55	Ononin	$C_{22}H_{22}O_9$	17.77	HR-MRM	429.1191	267.0663	267.0663	$C_{16}H_{11}O_4^-$	252.0428	$C_{15}H_8O_4^-$	100 / 52
56	*cis*-Resveratrol	$C_{14}H_{12}O_3$	18.00	HR-MRM	227.0714	143.0502	185.0608	$C_{12}H_9O_2^-$	143.0502	$C_{10}H_7O_1^-$	100 / 30

https://doi.org/10.3390/ph19101657

No.	Compound name	Molecular formula	Retention time, min	MS analysis type	Precursor ion, *m/z*	Measured ion *m/z*	MS/MS qualifier ion 1 *m/z*	MS/MS qualifier ion 1 formula	MS/MS qualifier ion 2 *m/z*	MS/MS qualifier ion 2 formula	Qualifier 1:2 ratio
57	Daidzein	$C_{15}H_{10}O_4$	18.40	HR-SIM	253.0506	253.0506	133.0295	$C_8H_5O_2^-$	223.0401	$C_{14}H_7O_3^-$	100 / 43
58	Quercetin	$C_{15}H_{10}O_7$	19.80	HR-SIM	301.0354	301.0354	151.0037	$C_7H_3O_4^-$	178.9986	$C_8H_3O_5^-$	100 / 36
59	Glycitein	$C_{16}H_{12}O_5$	20.10	HR-SIM	283.0612	283.0612	268.0377	$C_{15}H_8O_5^-$	239.0350	$C_{14}H_7O_4^-$	100 / 21
60	Luteolin	$C_{15}H_{10}O_6$	21.20	HR-SIM	285.0405	285.0405	133.0295	$C_8H_5O_2^-$	151.0037	$C_7H_3O_4^-$	100 / 82
61	Genistein	$C_{15}H_{10}O_5$	21.30	HR-SIM	269.0455	269.0455	133.0295	$C_8H_5O_2^-$	151.0037	$C_7H_3O_4^-$	100 / 60
62	Jaceosidin	$C_{17}H_{14}O_7$	21.80	HR-MRM	329.0667	271.0248	314.0432	$C_{14}H_7O_6^-$	299.0197	$C_{15}H_7O_7^-$	100 / 33
63	Kaempferol	$C_{15}H_{10}O_6$	22.70	HR-SIM	285.0405	285.0405	151.0037	$C_7H_3O_4^-$	257.0455	$C_{14}H_9O_5^-$	100 / 15
64	Coumestrol	$C_{15}H_8O_5$	23.20	HR-SIM	267.0299	267.0299	223.0401	$C_{14}H_7O_3^-$	252.0064	$C_{14}H_4O_5^-$	100 / 53
65	Apigenin	$C_{15}H_{10}O_5$	23.25	HR-SIM	269.0455	269.0455	117.0346	$C_8H_5O_1^-$	151.0037	$C_7H_3O_4^-$	100 / 52
66	Hispidulin	$C_{16}H_{12}O_6$	23.65	HR-MRM	299.0561	284.0326	284.0326	$C_{15}H_8O_6^-$	256.0377	$C_{14}H_8O_5^-$	100 / 20
67	Formononetin	$C_{16}H_{12}O_4$	24.80	HR-SIM	267.0663	267.0663	252.0428	$C_{15}H_8O_4^-$	133.0295	$C_8H_5O_2^-$	100 / 33
68	Eupatorin	$C_{18}H_{16}O_7$	26.30	HR-MRM	343.0823	313.0354	328.0589	$C_{17}H_{12}O_7^-$	313.0354	$C_{16}H_9O_7^-$	100 / 29
69	Eupatilin	$C_{18}H_{16}O_7$	26.72	HR-MRM	343.0823	313.0354	328.0589	$C_{17}H_{12}O_7^-$	313.0354	$C_{16}H_9O_7^-$	100 / 25
70	Pinocembrin	$C_{15}H_{12}O_4$	26.05	HR-SIM	255.0663	255.0663	213.0557	$C_{13}H_9O_3^-$	151.0037	$C_7H_3O_4^-$	100 / 71
71	Casticin	$C_{19}H_{18}O_8$	26.95	HR-MRM	373.0929	343.0459	358.0694	$C_{17}H_{11}O_8^-$	329.0667	$C_{17}H_{13}O_7^-$	100 / 20
72	Chrysin	$C_{15}H_{10}O_4$	27.35	HR-SIM	253.0506	253.0506	143.0502	$C_{10}H_7O_1^-$	209.0608	$C_{14}H_9O_2^-$	100 / 62
73	Galangin	$C_{15}H_{10}O_5$	27.90	HR-SIM	269.0455	269.0455	225.0557	$C_{14}H_9O_3^-$	151.0037	$C_7H_3O_4^-$	100 / 48
74	Acacetin	$C_{16}H_{12}O_5$	28.15	HR-MRM	283.0612	268.0377	268.0377	$C_{15}H_8O_5^-$	133.0295	$C_8H_5O_2^-$	100 / 32
75	Genkwanin	$C_{16}H_{12}O_5$	28.60	HR-MRM	283.0612	268.0377	268.0377	$C_{15}H_8O_5^-$	151.0037	$C_7H_3O_4^-$	100 / 44

HR-MS – high-resolution mass spectrometry; m/z – mass-to-charge ratio; min – minutes; MS – mass spectrometry; MS/MS – tandem mass spectrometry; HR-MRM—high-resolution multiple reaction monitoring; HR-SIM—high-resolution selected ion monitoring

Table S2. Calibration parameters, S/N-based detection thresholds, and quantitative performance at 0.05 µg/mL for phenolic compounds quantified using authentic standards.

		Calibration curve						
**No.**	**Compound name**	**Slope (peak area per µg/mL)**	**Intercept**	**Correlation coefficient (r)**	**Detection threshold at S/N ≥ 10 (µg/mL)**	**Intra-day precision at LOQ, RSD% (n=3)**	**Inter-day precision at LOQ, RSD% (n=3)**	**Accuracy at LOQ, % (*n*=3)**
1	Gallic acid	448122	-372046	0.9887	0.0190	7.1	8.52	110.03
2	Protocatechuic acid	307528	80349	0.9995	0.0227	5.69	8.15	101.01
3	Gentisic acid	320781	66226	0.9988	0.0167	7.82	11.71	105.09
4	Gallocatechin	184732	-30286	0.9970	0.0161	4.62	8.98	102.57
5	Epigallocatechin	187957	-36513	0.9965	0.0181	5.26	10.05	108.63
6	Salicin	62652	-20538	0.9980	0.0162	7.87	11.37	94.81
7	Procyanidin B3	111717	67624	0.9930	0.0233	4.16	11.34	108.99
8	Procyanidin B1	139284	-30210	0.9991	0.0139	5.98	11.29	105.14
9	Procyanidin B4	102463	64153	0.9892	0.0249	6.78	8.56	108.26
10	Procyanidin B2	115179	-68272	0.9910	0.0160	5.13	10.68	93.28
11	3-Hydroxybenzoic acid	240690	-143285	0.9902	0.0217	7.57	7.22	91.56
12	Esculin	1302010	-311322	0.9991	0.0152	6.33	11.9	91.79
13	Catechin	341136	-127026	0.9923	0.0171	7.75	8.84	91.53
14	Vanillic acid	204746	96770	0.9967	0.0208	4.8	8.94	105.76
15	Chlorogenic acid	386502	-293074	0.9893	0.0249	6.22	8.84	101.32
16	Caffeic acid	1022281	-278957	0.9984	0.0202	4.9	8.63	100.77
17	*cis*-Caffeic acid	841693	46877	0.9991	0.0175	4.97	10.98	101.33
18	Esculetin	1379928	-278650	0.9973	0.0157	7.7	8.32	95.12

https://doi.org/10.3390/ph19101657

				**Calibration curve**				
**No.**	**Compound name**	**Slope (peak area per µg/mL)**	**Intercept**	**Correlation coefficient (r)**	**Detection threshold at S/N ≥ 10 (µg/mL)**	**Intra-day precision at LOQ, RSD% (n=3)**	**Inter-day precision at LOQ, RSD% (n=3)**	**Accuracy at LOQ, % (*n*=3)**
19	4-*O*-Caffeoylquinic acid	180966	-8909	0.9985	0.0237	7.77	10.06	96.96
20	Syringic acid	222208	17227	0.9988	0.0138	7.88	10.17	91.44
21	Epigallocatechin gallate	174297	121870	0.9892	0.0196	6.49	9.85	110.39
22	Epicatechin	346355	99385	0.9975	0.0192	5.26	8.75	102.55
23	*p*-Coumaric acid	770615	-221593	0.9943	0.0151	4.52	10.67	98.91
24	*cis*-*p*-Coumaric acid	633411	293057	0.9980	0.0175	5.32	9.63	98.85
25	Procyanidin C1	111239	-26879	0.9930	0.0145	7.04	8.49	96.43
26	Genistin	377347	294	0.9985	0.0149	4.79	8.43	91.1
27	Umbeliferone	1919734	975492	0.9825	0.0163	4.79	8.87	100.93
28	Procyanidin A1	186599	-27721	0.9977	0.0256	5.48	7.48	90.07
29	Syringaldehyde	352494	-122299	0.9992	0.0169	6.18	6.18	91.45
30	Salicylic acid	961713	451812	0.9949	0.0187	4.01	6.13	93
31	Scopoletin	874889	261101	0.9969	0.0135	6.07	7.7	110.41
32	Ferulic acid	260087	207073	0.9894	0.0200	7.35	7.77	106.19
33	*cis*-Ferulic acid	318037	19291	0.9995	0.0186	4.84	9.27	103.73
34	Procyanidin A2	164926	-123219	0.9873	0.0148	6.76	11.71	104.54
35	Coniferyl aldehyde	1843387	-327612	0.9953	0.0185	7.79	11.47	104.83
36	*o*-Coumaric acid	424414	318500	0.9968	0.0216	7.54	9.19	92.78
37	Sinapaldehyde	601007	250153	0.9973	0.0238	6.79	6.09	88.16
38	Vitexin	319038	111210	0.9969	0.0167	4.73	11.71	100.44

https://doi.org/10.3390/ph19101657

		Calibration curve						
**No.**	**Compound name**	**Slope (peak area per µg/mL)**	**Intercept**	**Correlation coefficient (r)**	**Detection threshold at S/N ≥ 10 (µg/mL)**	**Intra-day precision at LOQ, RSD% (n=3)**	**Inter-day precision at LOQ, RSD% (n=3)**	**Accuracy at LOQ, % (*n*=3)**
39	Isovitexin	313078	105177	0.9968	0.0208	7.4	6.45	95.89
40	Vitexin-2-*O*-rhamnoside	415489	277387	0.9957	0.0190	4.78	6.19	96.96
41	Hyperoside	373392	159171	0.9943	0.0235	7.73	10.96	105.21
42	Isoquercitrin	411130	-298516	0.9895	0.0159	4.49	7.79	109
43	*trans*-Resveratrol	136609	9366	0.9998	0.0134	5.53	7.14	111.59
44	Quercetin-3-*O*-robinobioside	406852	9002	0.9995	0.0138	7.58	8.86	95.13
45	Rutin	407099	-5324	0.9996	0.0214	4.44	10.07	96.27
46	Ellagic acid	113870	26539	0.9986	0.0249	7.8	7.99	108.91
47	Rosmarinic acid	114797	12480	0.9979	0.0145	5.26	10.4	107.69
48	Myricetin	329374	-126579	0.9914	0.0216	5.25	10.45	98.19
49	Hesperidin	331903	-50042	0.9984	0.0208	4.56	10.12	108.79
50	Apigenin 7-glucoside	872200	91967	0.9968	0.0173	6.82	11.75	90.43
51	Quercitrin	332532	-16755	0.9978	0.0132	4.63	10.81	91.9
52	Fisetin	409440	215302	0.9840	0.0185	4.41	8.11	111.03
53	Kaempferol-3-*O*-glucoside	443172	243899	0.9922	0.0153	7.58	6.98	100.56
54	Quercetin-3-*O*-glucose-6-acetate	414791	-5984	0.9998	0.0163	6.26	6.83	103.22
55	Ononin	1745788	1219899	0.9978	0.0223	7.37	9.6	107.23
56	*cis*-Resveratrol	151023	-84988	0.9908	0.0191	4.87	8.11	104.72
57	Daidzein	2052860	-276049	0.9995	0.0163	6.48	6.77	109.46
58	Quercetin	455936	70525	0.9992	0.0172	6.27	6.47	111.54

https://doi.org/10.3390/ph19101657

				**Calibration curve**				
**No.**	**Compound name**	**Slope (peak area per µg/mL)**	**Intercept**	**Correlation coefficient (r)**	**Detection threshold at S/N ≥ 10 (µg/mL)**	**Intra-day precision at LOQ, RSD% (n=3)**	**Inter-day precision at LOQ, RSD% (n=3)**	**Accuracy at LOQ, % (*n*=3)**
59	Glycitein	1864089	565998	0.9940	0.0140	4.74	11.56	95.51
60	Luteolin	842579	91340	0.9999	0.0202	6.98	11.47	99.45
61	Genistein	2003952	229094	0.9944	0.0181	4.75	10.63	97.45
62	Jaceosidin	412485	-317168	0.9932	0.0208	6.51	7.56	101.96
63	Kaempferol	896926	203504	0.9994	0.0197	4.09	10.79	89.54
64	Coumestrol	1013803	303973	0.9990	0.0138	7.31	9.43	95.21
65	Apigenin	1101873	220949	0.9995	0.0187	4.75	8.22	97.95
66	Hispidulin	790907	-147302	0.9991	0.0186	4.84	8.83	102.62
67	Formononetin	2136453	-182704	0.9958	0.0196	5.37	11.58	95.5
68	Eupatorin	262068	51815	0.9980	0.0152	4.29	9.33	106.14
69	Eupatilin	299770	-56556	0.9985	0.0134	7.83	8.97	105.69
70	Pinocembrin	2410081	-986612	0.9988	0.0250	6.67	6.64	108.2
71	Casticin	319028	402601	0.9758	0.0164	4.84	9.18	105.3
72	Chrysin	1885991	-1047943	0.9985	0.0200	4.96	11.46	93.25
73	Galangin	1519700	-615078	0.9926	0.0147	5.26	6.47	105.69
74	Acacetin	1433754	1357131	0.9965	0.0199	6.45	6.44	98.78
75	Genkwanin	929197	-468131	0.9917	0.0156	4	10.07	96.23

r—correlation coefficient of the calibration curve; LOQ—lower limit of quantification; RSD—relative standard deviation; S/N—signal-to-noise ratio. Calibration curves were constructed over 0.05 – 50 µg/mL using linear regression. S/N-based detection thresholds were estimated at S/N ⩾ 10, whereas the LOQ (0.05 µg/mL) was established as the lowest calibration level meeting acceptance criteria for precision (RSD ⩽ 20%) and accuracy (80 – 120%).

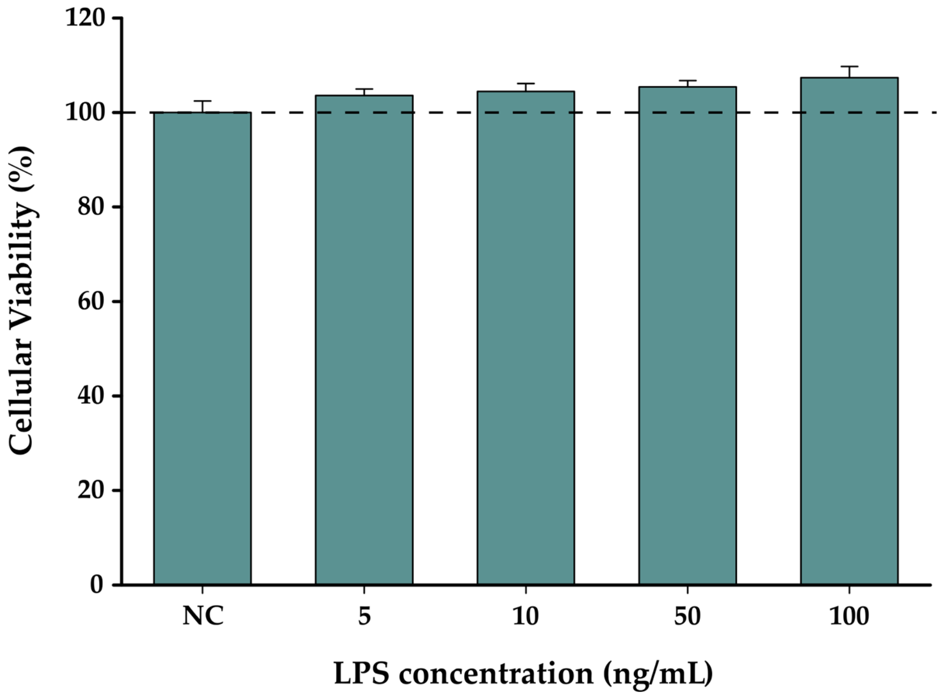

Figure S1. Effect of lipopolysaccharide (LPS) on cell viability. Cells were exposed to increasing concentrations of LPS (5, 10, 50, and 100 ng/mL) for 24 h, and cell viability was assessed using the Alamar Blue assay. NC denotes the negative control (untreated cells), and the dashed line indicates 100% viability. Results are expressed as a percentage of the negative control and presented as mean ± standard deviation (n = 3). No cytotoxic effect was observed at any of the tested concentrations.

https://doi.org/10.3390/ph19101657
